# What we learn about bipolar disorder from large‐scale neuroimaging: Findings and future directions from the ENIGMA Bipolar Disorder Working Group

**DOI:** 10.1002/hbm.25098

**Published:** 2020-07-29

**Authors:** Christopher R. K. Ching, Derrek P. Hibar, Tiril P. Gurholt, Abraham Nunes, Sophia I. Thomopoulos, Christoph Abé, Ingrid Agartz, Rachel M. Brouwer, Dara M. Cannon, Sonja M. C. de Zwarte, Lisa T. Eyler, Pauline Favre, Tomas Hajek, Unn K. Haukvik, Josselin Houenou, Mikael Landén, Tristram A. Lett, Colm McDonald, Leila Nabulsi, Yash Patel, Melissa E. Pauling, Tomas Paus, Joaquim Radua, Marcio G. Soeiro‐de‐Souza, Giulia Tronchin, Neeltje E. M. van Haren, Eduard Vieta, Henrik Walter, Ling‐Li Zeng, Martin Alda, Jorge Almeida, Dag Alnæs, Silvia Alonso‐Lana, Cara Altimus, Michael Bauer, Bernhard T. Baune, Carrie E. Bearden, Marcella Bellani, Francesco Benedetti, Michael Berk, Amy C. Bilderbeck, Hilary P. Blumberg, Erlend Bøen, Irene Bollettini, Caterina del Mar Bonnin, Paolo Brambilla, Erick J. Canales‐Rodríguez, Xavier Caseras, Orwa Dandash, Udo Dannlowski, Giuseppe Delvecchio, Ana M. Díaz‐Zuluaga, Danai Dima, Édouard Duchesnay, Torbjørn Elvsåshagen, Scott C. Fears, Sophia Frangou, Janice M. Fullerton, David C. Glahn, Jose M. Goikolea, Melissa J. Green, Dominik Grotegerd, Oliver Gruber, Bartholomeus C. M. Haarman, Chantal Henry, Fleur M. Howells, Victoria Ives‐Deliperi, Andreas Jansen, Tilo T. J. Kircher, Christian Knöchel, Bernd Kramer, Beny Lafer, Carlos López‐Jaramillo, Rodrigo Machado‐Vieira, Bradley J. MacIntosh, Elisa M. T. Melloni, Philip B. Mitchell, Igor Nenadic, Fabiano Nery, Allison C. Nugent, Viola Oertel, Roel A. Ophoff, Miho Ota, Bronwyn J. Overs, Daniel L. Pham, Mary L. Phillips, Julian A. Pineda‐Zapata, Sara Poletti, Mircea Polosan, Edith Pomarol‐Clotet, Arnaud Pouchon, Yann Quidé, Maria M. Rive, Gloria Roberts, Henricus G. Ruhe, Raymond Salvador, Salvador Sarró, Theodore D. Satterthwaite, Aart H. Schene, Kang Sim, Jair C. Soares, Michael Stäblein, Dan J. Stein, Christian K. Tamnes, Georgios V. Thomaidis, Cristian Vargas Upegui, Dick J. Veltman, Michèle Wessa, Lars T. Westlye, Heather C. Whalley, Daniel H. Wolf, Mon‐Ju Wu, Lakshmi N. Yatham, Carlos A. Zarate, Paul M. Thompson, Ole A. Andreassen, ENIGMA Bipolar Disorder Working Group

**Affiliations:** ^1^ Imaging Genetics Center, Mark and Mary Stevens Neuroimaging and Informatics Institute, Keck School of Medicine University of Southern California Los Angeles California USA; ^2^ Genentech, Inc. South San Francisco California USA; ^3^ Norwegian Centre for Mental Disorders Research (NORMENT), Institute of Clinical Medicine, University of Oslo Oslo Norway; ^4^ Division of Mental Health and Addicition, Oslo University Hospital Oslo Norway; ^5^ Department of Psychiatry Dalhousie University Halifax Nova Scotia Canada; ^6^ Faculty of Computer Science Dalhousie University Halifax Nova Scotia Canada; ^7^ Department of Clinical Neuroscience Karolinska Institutet Stockholm Sweden; ^8^ Department of Psychiatric Research Diakonhjemmet Hospital Oslo Norway; ^9^ Center for Psychiatric Research, Department of Clinical Neuroscience Karolinska Institutet Stockholm Sweden; ^10^ Department of Psychiatry, University Medical Center Utrecht Brain Center, University Medical Center Utrecht Utrecht University Utrecht The Netherlands; ^11^ Centre for Neuroimaging & Cognitive Genomics (NICOG), Clinical Neuroimaging Laboratory, NCBES Galway Neuroscience Centre, College of Medicine Nursing and Health Sciences National University of Ireland Galway Galway Ireland; ^12^ Department of Psychiatry University of California La Jolla California USA; ^13^ Desert‐Pacific MIRECC VA San Diego Healthcare San Diego California USA; ^14^ INSERM U955, team 15 “Translational Neuro‐Psychiatry” Créteil France; ^15^ Neurospin, CEA Paris‐Saclay, team UNIACT Gif‐sur‐Yvette France; ^16^ National Institute of Mental Health Klecany Czech Republic; ^17^ Norwegian Centre for Mental Disorders Research (NORMENT) Oslo University Hospital Oslo Norway; ^18^ APHP Mondor University Hospitals, DMU IMPACT Créteil France; ^19^ Department of Neuroscience and Physiology University of Gothenburg Gothenburg Sweden; ^20^ Department of Medical Epidemiology and Biostatistics Karolinska Institutet Stockholm Sweden; ^21^ Department for Psychiatry and Psychotherapy Charité Universitätsmedizin Berlin Berlin Germany; ^22^ Department of Neurology with Experimental Neurology Charité Universitätsmedizin Berlin Berlin Germany; ^23^ Bloorview Research Institute Holland Bloorview Kids Rehabilitation Hospital Toronto Ontario Canada; ^24^ Departments of Psychology and Psychiatry University of Toronto Toronto Ontario Canada; ^25^ Institut d'Investigacions Biomèdiques August Pi i Sunyer (IDIBAPS) Centro de Investigación Biomédica en Red de Salud Mental (CIBERSAM) Barcelona Spain; ^26^ Early Psychosis: Interventions and Clinical‐detection (EPIC) lab, Department of Psychosis Studies Institute of Psychiatry, Psychology and Neuroscience, King's College London London UK; ^27^ Stockholm Health Care Services Stockholm County Council Stockholm Sweden; ^28^ Mood Disorders Unit (GRUDA), Hospital das Clinicas HCFMUSP, Faculdade de Medicina Universidade de São Paulo São Paulo SP Brazil; ^29^ Department of Child and Adolescent Psychiatry/Psychology Erasmus Medical Center Rotterdam The Netherlands; ^30^ Barcelona Bipolar Disorders and Depressive Unit, Hospital Clinic, Institute of Neurosciences University of Barcelona Barcelona Spain; ^31^ College of Intelligence Science and Technology National University of Defense Technology Changsha China; ^32^ Dell Medical School The University of Texas at Austin Austin Texas USA; ^33^ FIDMAG Germanes Hospitalàries Research Foundation Barcelona Spain; ^34^ CIBERSAM Madrid Spain; ^35^ Milken Institute Center for Strategic Philanthropy Washington District of Columbia USA; ^36^ Department of Psychiatry and Psychotherapy, Medical Faculty Technische Universität Dresden Dresden Germany; ^37^ Department of Psychiatry University of Münster Münster Germany; ^38^ Department of Psychiatry The University of Melbourne Melbourne Victoria Australia; ^39^ The Florey Institute of Neuroscience and Mental Health The University of Melbourne Melbourne Victoria Australia; ^40^ Department of Psychiatry and Biobehavioral Sciences, Semel Institute for Neuroscience and Human Behavior University of California Los Angeles California USA; ^41^ Department of Psychology University of California Los Angeles California USA; ^42^ Section of Psychiatry, Department of Neurosciences, Biomedicine and Movement Sciences University of Verona Verona Italy; ^43^ Vita‐Salute San Raffaele University Milan Italy; ^44^ Division of Neuroscience, Psychiatry and Psychobiology Unit IRCCS San Raffaele Scientific Institute Milan Italy; ^45^ Department of Pathophysiology and Transplantation University of Milan Milan Italy; ^46^ IMPACT Institute – The Institute for Mental and Physical Health and Clinical Translation, School of Medicine, Barwon Health Deakin University Geelong Victoria Australia; ^47^ The National Centre of Excellence in Youth Mental Health, Centre for Youth Mental Health, Florey Institute for Neuroscience and Mental Health and the Department of Psychiatry, The University of Melbourne Orygen Melbourne Victoria Australia; ^48^ P1vital Ltd Wallingford UK; ^49^ Department of Psychiatry University of Oxford Oxford UK; ^50^ Mood Disorders Research Program Yale School of Medicine New Haven Connecticut USA; ^51^ Psychosomatic and CL Psychiatry Oslo University Hospital Oslo Norway; ^52^ Department of Neurosciences and Mental Health Fondazione IRCCS Ca' Granda Ospedale Maggiore Policlinico Milan Italy; ^53^ Department of Radiology Centre Hospitalier Universitaire Vaudois (CHUV) Lausanne Switzerland; ^54^ Signal Processing Lab (LTS5), École Polytechnique Fédérale de Lausanne Lausanne Switzerland; ^55^ MRC Centre for Neuropsychiatric Genetics and Genomics Cardiff University Cardiff UK; ^56^ Melbourne Neuropsychiatry Centre, Department of Psychiatry University of Melbourne and Melbourne Health Melbourne Victoria Australia; ^57^ Brain, Mind and Society Research Hub, Turner Institute for Brain and Mental Health, School of Psychological Sciences Monash University Clayton Victoria Australia; ^58^ Research Group in Psychiatry GIPSI, Department of Psychiatry Faculty of Medicine, Universidad de Antioquia Medellín Colombia; ^59^ Department of Psychology, School of Social Sciences and Arts City, University of London London UK; ^60^ Department of Neuroimaging, Institute of Psychiatry, Psychology & Neuroscience King's College London London UK; ^61^ Department of Neurology Oslo University Hospital Oslo Norway; ^62^ Institute of Clinical Medicine University of Oslo Oslo Norway; ^63^ Center for Neurobehavioral Genetics Los Angeles California USA; ^64^ Greater Los Angeles Veterans Administration Los Angeles California USA; ^65^ Centre for Brain Health University of British Columbia Vancouver British Columbia Canada; ^66^ Department of Psychiatry Icahn School of Medicine at Mount Sinai New York New York USA; ^67^ Neuroscience Research Australia Randwick New South Wales Australia; ^68^ School of Medical Sciences University of New South Wales Sydney New South Wales Australia; ^69^ Department of Psychiatry Boston Children's Hospital and Harvard Medical School Boston Massachusetts USA; ^70^ School of Psychiatry University of New South Wales Sydney New South Wales Australia; ^71^ Department of General Psychiatry Heidelberg University Heidelberg Germany; ^72^ Department of Psychiatry, University Medical Center Groningen University of Groningen Groningen The Netherlands; ^73^ Department of Psychiatry Service Hospitalo‐Universitaire, GHU Paris Psychiatrie & Neurosciences Paris France; ^74^ Université de Paris Paris France; ^75^ Neuroscience Institute University of Cape Town Cape Town South Africa; ^76^ Department of Psychiatry and Mental Health University of Cape Town Cape Town South Africa; ^77^ Core‐Facility Brainimaging, Faculty of Medicine University of Marburg Marburg Germany; ^78^ Department of Psychiatry and Psychotherapy Philipps‐University Marburg Marburg Germany; ^79^ Department of Psychiatry, Psychosomatic Medicine and Psychotherapy Goethe University Frankfurt Frankfurt Germany; ^80^ Laboratory of Psychiatric Neuroimaging (LIM‐21), Departamento e Instituto de Psiquiatria Hospital das Clinicas HCFMUSP, Faculdade de Medicina, Universidade de São Paulo São Paulo SP Brazil; ^81^ Mood Disorders Program Hospital Universitario Trastorno del Ánimo Medellín Colombia; ^82^ Experimental Therapeutics and Molecular Pathophysiology Program, Department of Psychiatry UTHealth, University of Texas Houston Texas USA; ^83^ Hurvitz Brain Sciences Sunnybrook Research Institute Toronto Ontario Canada; ^84^ Department of Medical Biophysics University of Toronto Toronto Ontario Canada; ^85^ University of Cincinnati Cincinnati Ohio USA; ^86^ Universidade de São Paulo São Paulo SP Brazil; ^87^ MEG Core Facility Bethesda Maryland USA; ^88^ UCLA Center for Neurobehavioral Genetics Los Angeles California USA; ^89^ Department of Psychiatry Erasmus Medical Center, Erasmus University Rotterdam The Netherlands; ^90^ Department of Mental Disorder Research National Institute of Neuroscience, National Center of Neurology and Psychiatry Tokyo Japan; ^91^ Department of Psychiatry University of Pittsburgh Pittsburgh Pennsylvania USA; ^92^ Research Group Instituto de Alta Tecnología Médica (IATM) Medellín Colombia; ^93^ University of Grenoble Alpes CHU Grenoble Alpes Grenoble France; ^94^ INSERM U1216 ‐ Grenoble Institut des Neurosciences La Tronche France; ^95^ Department of Psychiatry Amsterdam UMC, location AMC Amsterdam The Netherlands; ^96^ Department of Psychiatry Radboud University Medical Center Nijmegen The Netherlands; ^97^ Donders Institute for Brain, Cognition and Behavior Radboud University Nijmegen The Netherlands; ^98^ Department of Psychiatry University of Pennsylvania Perelman School of Medicine Philadelphia Pennsylvania USA; ^99^ West Region, Institute of Mental Health Singapore Singapore; ^100^ Yong Loo Lin School of Medicine National University of Singapore Singapore Singapore; ^101^ Center of Excellent on Mood Disorders UTHealth Houston Houston Texas USA; ^102^ Department of Psychiatry and Behavioral Sciences UTHealth Houston Houston Texas USA; ^103^ SAMRC Unit on Risk & Resilience in Mental Disorders University of Cape Town Cape Town South Africa; ^104^ PROMENTA Research Center, Department of Psychology University of Oslo Oslo Norway; ^105^ Papanikolaou General Hospital Thessaloniki Greece; ^106^ Laboratory of Mechanics and Materials School of Engineering, Aristotle University Thessaloniki Greece; ^107^ Department of Psychiatry Amsterdam UMC Amsterdam The Netherlands; ^108^ Department of Neuropsychology and Clinical Psychology Johannes Gutenberg‐University Mainz Mainz Germany; ^109^ Department of Psychology University of Oslo Oslo Norway; ^110^ Norwegian Centre for Mental Disorders Research (NORMENT), Department of Mental Health and Addiction Oslo University Hospital Oslo Norway; ^111^ Centre for Clinical Brain Sciences University of Edinburgh Edinburgh UK; ^112^ Department of Psychiatry University of British Columbia Vancouver British Columbia Canada; ^113^ Chief Experimental Therapeutics & Pathophysiology Branch Bethesda Maryland USA; ^114^ Intramural Research Program National Institute of Mental Health Bethesda Maryland USA

**Keywords:** bipolar disorder, cortical surface area, cortical thickness, ENIGMA, mega‐analysis, meta‐analysis, MRI, neuroimaging, psychiatry, volume

## Abstract

MRI‐derived brain measures offer a link between genes, the environment and behavior and have been widely studied in bipolar disorder (BD). However, many neuroimaging studies of BD have been underpowered, leading to varied results and uncertainty regarding effects. The Enhancing Neuro Imaging Genetics through Meta‐Analysis (ENIGMA) Bipolar Disorder Working Group was formed in 2012 to empower discoveries, generate consensus findings and inform future hypothesis‐driven studies of BD. Through this effort, over 150 researchers from 20 countries and 55 institutions pool data and resources to produce the largest neuroimaging studies of BD ever conducted. The ENIGMA Bipolar Disorder Working Group applies standardized processing and analysis techniques to empower large‐scale meta‐ and mega‐analyses of multimodal brain MRI and improve the replicability of studies relating brain variation to clinical and genetic data. Initial BD Working Group studies reveal widespread patterns of lower cortical thickness, subcortical volume and disrupted white matter integrity associated with BD. Findings also include mapping brain alterations of common medications like lithium, symptom patterns and clinical risk profiles and have provided further insights into the pathophysiological mechanisms of BD. Here we discuss key findings from the BD working group, its ongoing projects and future directions for large‐scale, collaborative studies of mental illness.

## Graphical abstract

This review discusses the major challenges facing neuroimaging research of bipolar disorder and highlights the major accomplishments, ongoing challenges and future goals of the ENIGMA Bipolar Disorder Working Group.

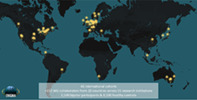

## 1 INTRODUCTION

### 1.1 Overview

Bipolar disorder (BD) is a severe mental disorder characterized by episodic alterations in mood and activity levels including depression, hypomania and mania. It is a leading cause of disability and affects ~1% of the world's population (Merikangas et al., 2011; Vieta et al., 2018). As a chronic illness, BD can lead to long‐term functional impairments and reduced quality of life for both patients and caregivers (Oldis et al., 2016; Perlick, Rosenheck, Clarkin, Raue, & Sirey, 2001; Vigo, Thornicroft, & Atun, 2016), which confer significant societal costs (Ekman, Granstrom, Omerov, Jacob, & Landen, 2013). Challenges remain in identifying robust and reproducible biomarkers to better understand the neurobiology, nosology, diagnosis and targeted treatments that are needed to improve patient outcomes in BD. Mental health professionals continue to rely on a phenomenology‐based diagnostic system as opposed to validated biological markers^1^ to diagnose and treat BD. This is reflected in current diagnostic classification systems, namely the DSM‐5 (APA, 2013) and ICD‐10 (WHO, 1993; https://apps.who.int/iris/handle/10665/246208), which use the number and profile of symptoms to delineate the unique and overlapping clinical features of mental disorders, and allow individuals to be categorized based on threshold criteria (BD diagnosis criteria provided in Supplemental Materials). While such approaches have improved the reliability of diagnosing BD, misdiagnosis remains common, and hence biomarkers such as those recently accepted for other disorders such as Alzheimer's disease (Jack Jr. et al., 2018) are urgently needed for BD. To date, there are still no valid biomarkers for the diagnosis of any mental disorder, including BD (Quevedo & Yatham, 2018; Vieta et al., 2018; Vieta & Phillips, 2007).

**FIGURE 1:**
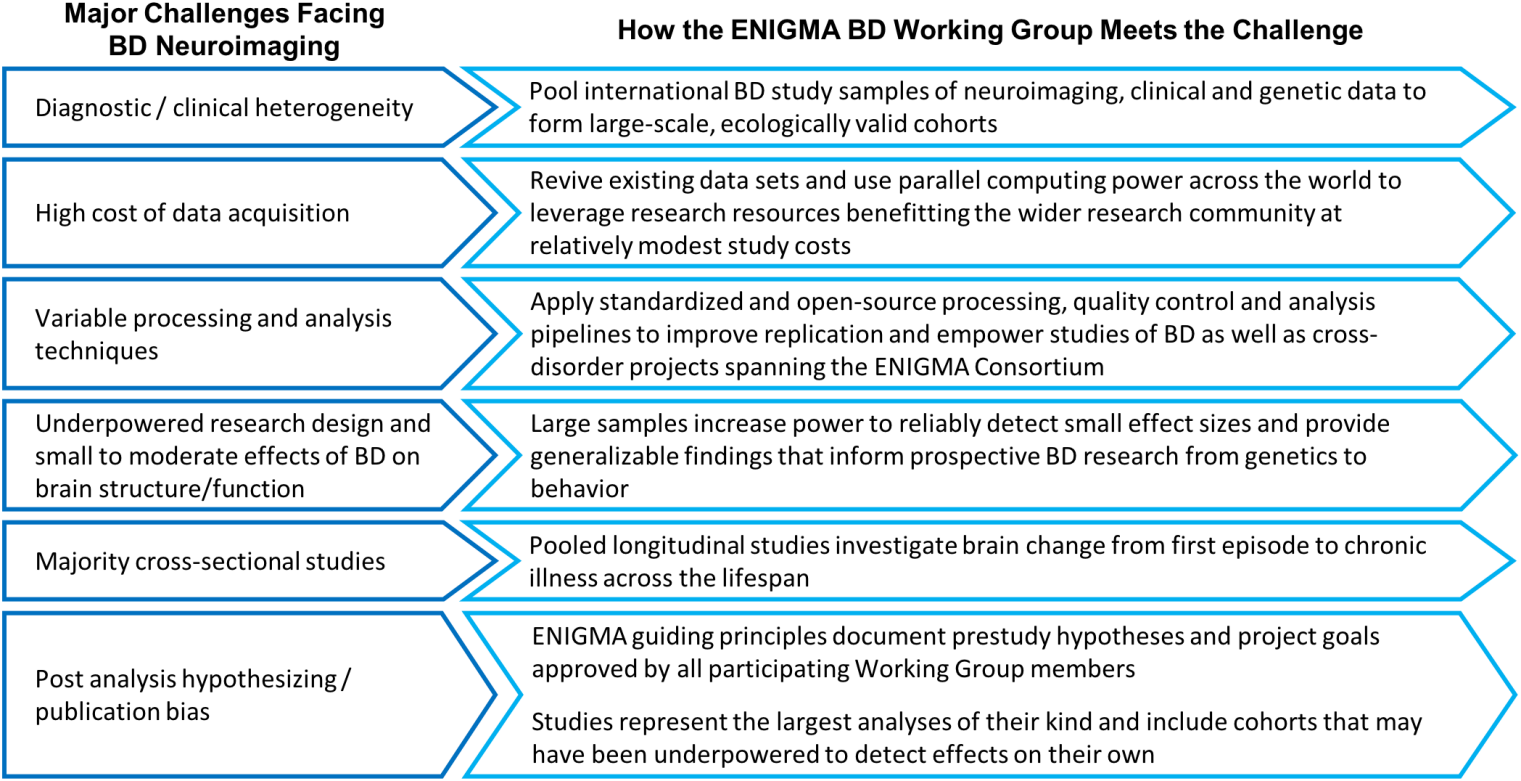
Major challenges facing neuroimaging studies of BD and how the ENIGMA BD Working Group meets these challenges

Non‐invasive, in vivo measures of brain structure and function derived from magnetic resonance imaging (MRI) have shed light on the underlying brain alterations associated with BD. However, factors such as clinical heterogeneity, high costs of data acquisition, variable processing and analysis protocols, underpowered and generally cross‐sectional research designs, post analysis hypothesizing and publication bias have resulted in variable findings and hindered the discovery of validated biomarkers (Figure 1).

### 1.2 Key challenges facing the use of neuroimaging to study bipolar disorder

Neuroimaging has been used as a powerful, non‐invasive tool to study mental disorders such as BD for several decades, ever since early studies revealing enlarged ventricles in patients with schizophrenia (Johnstone, Crow, Frith, Husband, & Kreel, 1976). A key goal of psychiatric neuroimaging research is to use MRI measures of brain structure and function as a link between phenomenology, course, outcome, treatment response, genetics and behavior. Unlike behavioral outcome measures, MRI‐derived brain measures have been proposed to serve as an “endophenotype” – or a marker more closely related to the underlying biology of a disorder. Such markers may have a simpler genetic architecture or be more tractable to study compared to behavioral phenotypes. There is mounting evidence that these in vivo brain endophenotypes may help define the causal pathways between heterogeneous behavioral alterations and genetic factors associated with a given mental disorder (Bigos & Weinberger, 2010; Glahn, Thompson, & Blangero, 2007; Gottesman & Shields, 1973, 1976; Holland et al., 2020; Le & Stein, 2019).

A large body of both structural and functional neuroimaging research has associated BD with abnormalities in the neural circuitry of emotion and reward processing (Phillips & Swartz, 2014). Structural studies have shown BD‐related variation in cortical regions including prefrontal, anterior temporal and insula cortices (Ganzola & Duchesne, 2017; Hajek et al., 2013; Rimol et al., 2012) as well as alterations in subcortical structures such as the hippocampus, thalamus and amygdala in individuals with BD compared to healthy controls (HC) (Hajek et al., 2009; Hajek, Kopecek, Hoschl, & Alda, 2012; Rimol et al., 2010). Studies of white matter in individuals with BD using diffusion MRI (dMRI) have found disruptions in fronto‐limbic white matter (WM) regions important to emotion regulation and reward processing (Phillips & Swartz, 2014). Functional neuroimaging studies have tied altered prefrontal, amygdala, temporal and ventral striatum activity during emotion and reward tasks to BD (Phillips & Swartz, 2014; Sepede et al., 2012, 2015, 2020).

However, while prior meta‐analyses have found relative consensus across studies (Ganzola & Duchesne, 2017), conflicting results are not uncommon in neuroimaging studies of BD. For example, reports of both larger and smaller structural volumes in participants with BD versus HC are widely cited in the literature (Phillips & Swartz, 2014). Such discrepancies can be traced to a core set of challenges facing neuroimaging studies of BD (Figure 1; see Supplemental Materials for further details on these challenges).

First, BD is a broad diagnostic category with varied clinical characteristics likely including related underlying subtypes (i.e., biotypes that subdivide or stratify conventional diagnostic categories). These subtypes likely vary in underlying genetic and environmental risk, treatment response, psychiatric/medical comorbidity, clinical course, prognosis and pathophysiology (Duffy, Vandeleur, Heffer, & Preisig, 2017). Even the most carefully designed studies of BD will likely include participant heterogeneity, as we do not fully understand the underlying neurobiological stratification. We discuss the challenges of linking current categorical diagnostic constructs of BD to underlying biological measures in the Supplemental Materials. Study participant heterogeneity in key variables affecting brain measures (e.g., diagnostic method, age at scan, age of onset, illness duration, medication status, etc.) adds noise to the already complicated task of detecting the subtle effects of the disorder on MRI‐derived brain metrics. Second, the high cost of MRI data collection can restrict study sample sizes (most studies collect data from fewer than 100 participants), which may limit the statistical power to detect neurobiological effects or model complex factors modulating BD symptoms. Third, variable processing and analysis techniques for MRI make results hard to compare across studies (Botvinik‐Nezer et al., 2020). The power of traditional literature‐based meta‐analyses in BD neuroimaging studies is somewhat limited by a lack of such standardized techniques (see the following section for more detail). Fourth, most research studies are cross‐sectional, which limits analyses to static brain traits as opposed to potential brain alterations over the course of illness in BD. Fifth, underpowered and heterogeneous study samples may yield false positives or overestimate effects – a situation known as the “winner's curse” (Button et al., 2013). Lastly, many studies suffer from post analysis hypothesizing or publication bias. All these challenges degrade the reliability of findings, mandating the design of more replicable and generalizable studies.

Under current diagnostic criteria, BD patient heterogeneity (Charney et al., 2017; Stahl et al., 2019; Wolfers et al., 2018) can affect study power depending on sample size (Button et al., 2013). In smaller samples, patient heterogeneity can dilute the overall effect of interest, either making it harder to detect subtle BD alterations or overestimating true BD effects. In larger consortium studies with increased statistical power, patient heterogeneity can lead to a more “ecologically valid” sample that represents a larger fraction of the patient space. While this heterogeneity in large samples can lead to more replicable findings that apply more widely to BD in the general population, subtle biological effects relevant to particular patient subgroups may be obscured, requiring data‐driven, post‐hoc stratification. Smaller, well‐controlled studies that are able to implement deeper phenotyping and novel analysis methods may be equipped to isolate the underlying pathophysiology associated with particular patient subgroups or symptom profiles, as long as the sample sizes are still adequately powered to detect the effects.

Current structural and functional neuroimaging measures may only account for a limited proportion of the overall variance in a complex phenotypic trait such as BD diagnosis (Paulus & Thompson, 2019). Larger research samples may overcome the power limitations of smaller studies and improve sensitivity to subtle brain signatures (Westlye, Alnaes, van der Meer, Kaufmann, & Andreassen, 2019). They may also offer a greater opportunity to model factors that contribute to complex phenotypes such as BD. For example, some medications commonly used to treat BD have shown morphometric effects on MRI‐derived brain metrics (Abe et al., 2015; Abramovic et al., 2016; Hajek et al., 2014; Haukvik et al., 2020; Lyoo et al., 2010; Moore et al., 2009; Moore, Bebchuk, Wilds, Chen, & Manji, 2000; Sarrazin et al., 2019), including gray matter increases with lithium and atrophic effects of anticonvulsants (Hibar et al., 2018). These medication‐related brain changes may be critical in the treatment of BD, but they may also make brain alterations in the disorder harder to detect, as most are highly confounded with illness status or severity. The modeling of complex factors, such as medication history, can be greatly improved in larger, pooled study samples.

These core challenges have contributed to the “replication crisis” affecting much of biomedical research (Button et al., 2013; Dumas‐Mallet, Button, Boraud, Gonon, & Munafo, 2017; Ioannidis, 2008, 2011, 2017). To address these challenges, and to contribute to a recent shift in psychiatry toward large consortium efforts, the ENIGMA Bipolar Disorder Working Group was formed to pool data, expertise and computational resources to discover factors that reliably affect brain structure and function in BD.

Importantly, clinical symptoms (either categorical or dimensional) remain the most useful metrics available to clinicians treating patients with BD. While some of the research tools presented in this article may one day improve BD care, these tools and methods must be rigorously validated and standardized if they are ever to be truly useful in a clinical setting. In the following sections, we discuss how large‐scale efforts provide a path forward, and serve as an example of the power of team science in pooling existing data and expertise to tackle the core challenges facing BD research and treatment.

### 1.3 The ENIGMA consortium: Large‐scale, collaborative studies of brain structure and function

An increasing number of common variants throughout the human genome have been associated with risk for BD. The Psychiatric Genomics Consortium (PGC) spearheaded a global effort to discover these risk loci by coordinating large‐scale psychiatric genetics studies (https://www.med.unc.edu/pgc/). Many early genetic studies prior to the PGC found associations between polymorphisms in candidate genes and brain measures that later failed to replicate in larger independent samples (Farrell et al., 2015). Genome‐wide analyses have now revealed hundreds of common genetic variants that are reliably associated with psychiatric disorders (Bipolar Disorder and Schizophrenia Working Group of the Psychiatric Genomics Consortium, 2018; Schizophrenia Working Group of the Psychiatric Genomics Consortium, 2014; Stahl et al., 2019; Wray et al., 2018), as well as discovered significant genetic overlap between major disorders (Brainstorm et al., 2018; Gandal et al., 2018).

As the PGC has done for genetics, large‐scale, collaborative neuroimaging studies offer the power to answer new questions and address prior inconsistencies in the literature. The ENIGMA Consortium, launched in 2009, aimed to identify genetic variants that are consistently associated with brain structure and function by performing genome‐wide association studies (GWAS) on measures from brain MRI. To overcome the statistical power limitations of GWAS (as most genetic polymorphisms associated with brain measures account for less than 1% of the overall variance in any given brain measure), the initial ENIGMA projects recruited samples with both MRI and genetic data and implemented standardized processing and analysis methods. These protocols enabled highly powered, prospective meta‐analyses on a scale not previously possible (i.e., 99% power to detect genetic loci explaining at least 1% of the variance in a given brain trait). These initial studies identified new genetic variants associated with variability in brain volumes such as the hippocampus (Hibar et al., 2015; Stein et al., 2012). More recently, collaborations between ENIGMA and other large‐scale international consortia, such as CHARGE, were able to replicate initial findings in larger independent samples and provided new insights into the genetic mechanisms influencing brain structure (Adams et al., 2016; Grasby et al., 2020; Hibar et al., 2017; Satizabal et al., 2019), function (Smit et al., 2018), and development (Brouwer et al., 2017).

The success of the initial ENIGMA multi‐site GWAS led to the formation of over 50 collaborative ENIGMA Working Groups developing or using standardized protocols and studying a wide range of neurodegenerative, neurodevelopmental and psychiatric disorders. Working in parallel, and sharing standardized tools, the ENIGMA clinical working groups published the largest neuroimaging studies of BD (Hibar et al., 2016; Hibar et al., 2018), MDD (Schmaal et al., 2016; Schmaal et al., 2017), schizophrenia (SCZ) (van Erp et al., 2016; van Erp et al., 2018), obsessive compulsive disorder (OCD) (Boedhoe et al., 2017; Boedhoe et al., 2018), attention deficit hyperactivity disorder (ADHD) (Hoogman et al., 2017, 2019), autism spectrum disorder (ASD) (van Rooij et al., 2018), epilepsy (Whelan et al., 2018), substance use disorders (Mackey et al., 2019), PTSD (Dennis et al., 2019; Logue et al., 2018) and 22q11.2 deletion syndrome (Ching et al., 2020; Sun et al., 2018; Villalon‐Reina et al., 2019). A more thorough review of efforts across the ENIGMA Consortium may be found in several recent articles (Bearden & Thompson, 2017; Thompson et al., 2014; Thompson et al., 2020).

The ENIGMA Consortium offers a number of advantages compared to previous smaller‐scale neuroimaging studies. ENIGMA takes advantage of existing data sets, reviving smaller samples that have often concluded data collection and primary publications. To date, the ENIGMA consortium has incorporated over 70,000 scans from a range of disorders and healthy individuals. ENIGMA currently includes over 2,000 scientists from over 340 institutions, spanning 45 countries. By taking advantage of existing data and using parallel computing power across the world, ENIGMA has leveraged research resources that benefit the wider research community at a relatively modest study cost.

In the conventional, retrospective, literature‐based meta‐analysis, summary statistics including effect sizes, confidence intervals and standard errors are extracted from published studies (which have often used different processing and analysis pipelines). These are then combined mathematically to estimate an overall effect (e.g., regional brain volume differences between BD and HC). A key strength of the ENIGMA approach over more traditional meta‐analyses is the implementation of standardized processing and analysis protocols to perform coordinated, prospective meta‐ and mega‐analyses. These protocols, which are publicly available (http://enigma.ini.usc.edu/protocols), serve several purposes: 1) ENIGMA‐standardized protocols make it possible to efficiently and consistently extract measures from MRI data and to perform robust quality assessment and statistical modeling across tens to hundreds of international research centers with varying neuroimaging expertise, 2) standardized processing and analysis leads to more unbiased investigations of brain measures not possible via traditional meta‐analyses that combine published effect sizes derived from varied processing and analysis protocols (e.g., regions of interest combined with whole brain analyses, mass‐univariate combined with multivariate analyses, etc.), 3) pooling data can overcome publication bias and boost statistical power by including cohorts that may have been underpowered on their own to detect effects on particular brain measures or associations with symptom constructs, 4) harmonized protocols using standardized and publicly available pipelines promote “open science” by increasing transparency and encouraging replication, 5) it makes large‐scale, post‐hoc subgroup explorations possible and lastly, 6) it allows for the direct comparison of large‐scale, standardized data across ENIGMA Working Groups to better understand common and distinct patterns that span traditional diagnostic boundaries.

Importantly, and as previously mentioned, large‐scale ENIGMA BD Working Group efforts do not replace well‐designed, smaller‐scale neuroimaging studies, but rather complement one another. While the initial ENIGMA studies were well powered to capture generalizable effects across study samples, they can miss subtle, subtype‐specific effects that may be better captured by smaller well‐controlled studies focusing on particular subtypes or symptom patterns in BD, or with targeted deeper phenotyping not economically feasible in large‐scale studies. While the large samples pooled through ENIGMA increase sensitivity to smaller effect sizes, and are arguably more generalizable to the wider population, potential lurking confounds or artifacts (e.g., head motion see Pardoe, Hiess, & Kuzniecky, 2016; Yao et al., 2017) that may span independently collected study samples must be carefully considered and modeled.

Lastly, the international coordination of ENIGMA studies requires consensus and cooperation across a large number of centers worldwide. In our experience, one of the more important steps to starting a successful ENIGMA Working Group is to be pragmatic about the initial studies carried out by the group. Focusing early efforts on a core set of available variables and feasibly derived brain measures lowers the bar for participation and helps to incorporate the greatest number of Working Group members. These early studies, while not necessarily applying the most novel of brain metrics (e.g., volumes) or analysis models (e.g., general linear models) are more feasible to accomplish while simultaneously providing important consensus building findings in the literature. The trust and infrastructure formed through these initial pragmatic studies and publications helps build momentum toward more ambitious projects using more advanced metrics (e.g., vertex‐wise measures, connectivity measures using graph theory, etc.) and more advanced analyses (e.g., multivariate analysis, machine learning, structural covariance analysis, etc.).

## 2 THE ENIGMA BIPOLAR DISORDER WORKING GROUP

**FIGURE 2:**
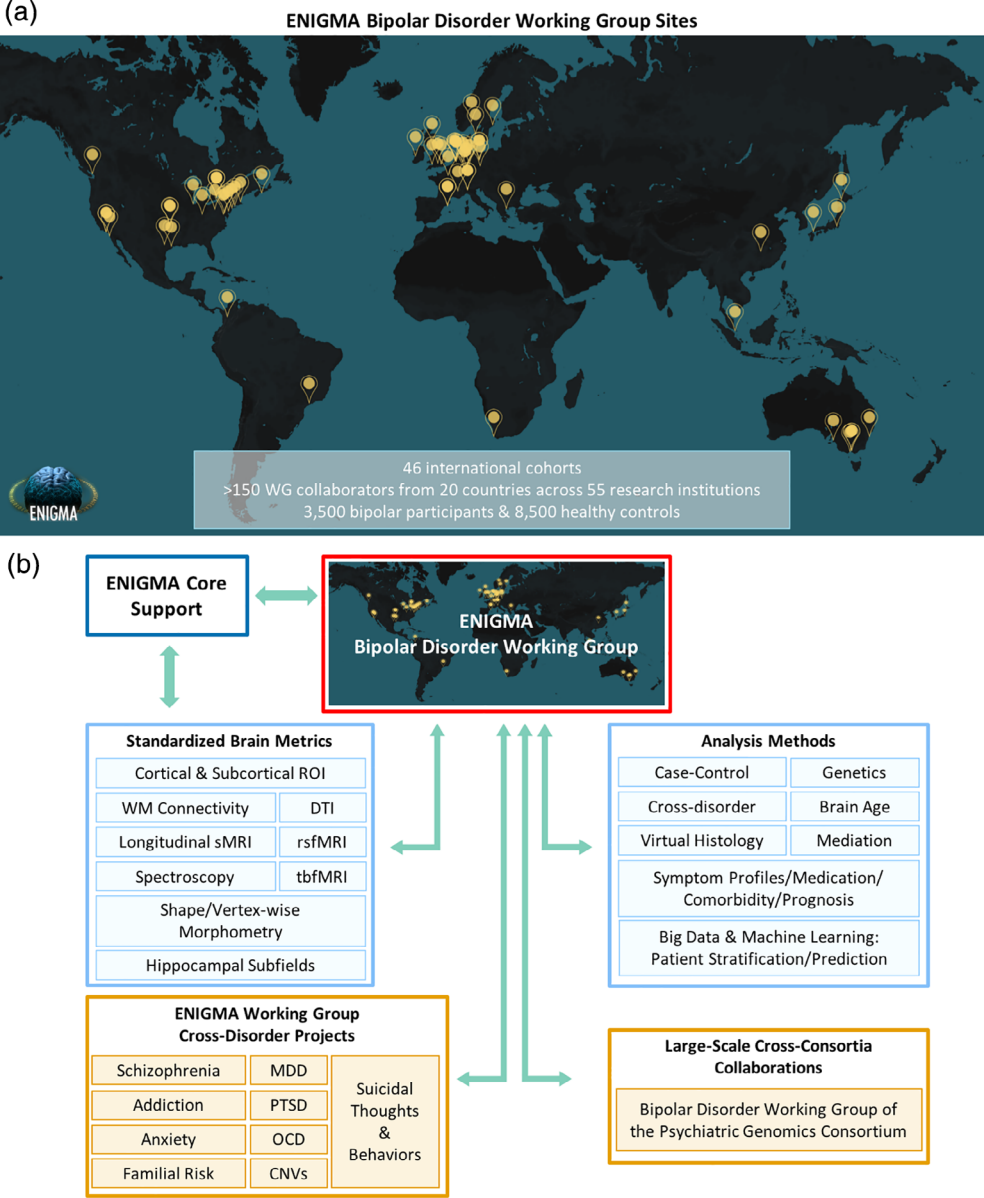
(a) ENIGMA Bipolar Disorder Working Group sites across the world including over 150 researchers from 20 countries and 55 institutions. (b) Schematic of ENIGMA Bipolar Disorder Working Group as it fits into the larger ENIGMA Consortium network. rsfMRI, resting‐state functional MRI; tbfMRI, task‐based functional MRI; WM, white matter; DTI, diffusion tensor imaging; MDD, major depressive disorder; PTSD, post‐traumatic stress disorder; OCD, obsessive–compulsive disorder; CNVs, copy number variants; Familial Risk, relatives of individuals with psychiatric illness (including bipolar disorder and schizophrenia)

The ENIGMA Bipolar Disorder Working Group was formed in 2012 to address some of the core limitations in BD research and foster collaborative discoveries using the wider ENIGMA Consortium research model. The Working Group consists of an international team of over 150 clinicians, neuroscientists, bioengineers, and geneticists who pool research resources from 20 countries and 55 institutions to conduct large‐scale neuroimaging studies of BD. The group has combined multimodal neuroimaging data from 46 study cohorts which include ~3,500 BD participants and ~ 8,500 healthy controls, making it the largest neuroimaging consortium effort to study BD (Figure 2 **)**. In the following sections we discuss the Bipolar Disorder Working Group and its contributions to understanding brain alterations in BD, including guiding principles, current findings, ongoing projects and future directions that aim to advance neurobiological research of BD and other mental disorders.

### 2.1 Goals and guiding principles

The primary research goals of the Bipolar Disorder Working Group are to:

1. Make reliable discoveries that improve our understanding of the pathophysiological mechanisms of BD.
2. Provide clinically relevant information to help improve BD nosology, diagnosis, mechanistically‐directed interventions and treatment outcomes.

To accomplish these goals, the Bipolar Disorder Working Group implemented ENIGMA‐standardized protocols using open‐source and widely available processing platforms such as FreeSurfer (Fischl et al., 2002) and FSL (Smith et al., 2006), as well as tools specifically developed for large‐scale, multisite projects **(**Figure 2 **)**.

**FIGURE 3:**
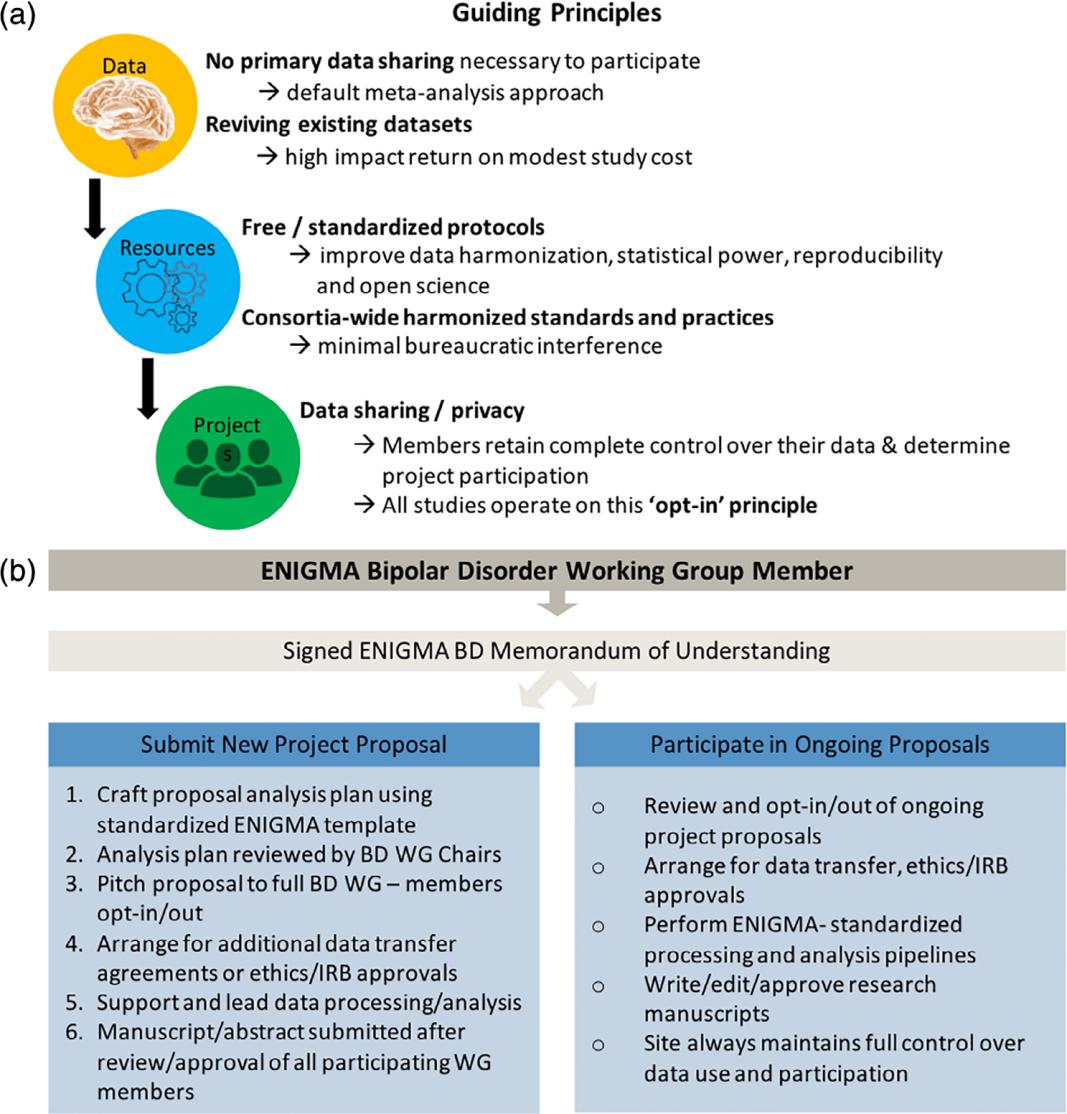
(a) Outline of the ENIGMA BD Working Group guiding principles. (b) Flow diagram showing working group logistics including memorandum of understanding, participation in and development of new research proposals, data sharing, etc. Ethics/IRB: The ENIGMA BD Working group is experienced with navigating international research ethics and institutional review boards, which may require additional approval depending on project specifics. More information on the ENIGMA BD Working group including the Memorandum of Understanding can be found online (http://enigma.ini.usc.edu/ongoing/enigma‐bipolar‐working‐group/)

The BD Working Group operates under a set of simple guiding principles (Figure 3). First, primary data sharing is not required to participate in the working group; meta‐analysis is always the encouraged/default analysis framework. This helps garner the greatest number of participating samples and mitigates many data sharing challenges (Zugman et al., 2020). Working Group projects operate on an “opt‐in” principle, where members retain full ownership and control over their data and are free to decide which project proposals they wish to participate in. Furthermore, members can withdraw their data or resources from a project at any time prior to publication.

New Working Group members are asked to sign a Memorandum of Understanding (MOU) that has been standardized across the ENIGMA Working Groups and sets a basic framework to protect participating sites' data privacy, facilitate data sharing, encourage academic productivity, ensure appropriate publication credit and authorship**.** It also provides a system to track and archive data, analyses and publications related to the BD Working Group (http://enigma.ini.usc.edu/ongoing/enigma-bipolar-working-group/ Figure 3). The Working Group's scientific initiatives and basic policies are overseen by two Chairs, Dr. Ole A. Andreassen and Dr. Christopher R. K. Ching, who are responsible for ensuring that working group studies are carried out in accordance with appropriate ethical guidelines.

Working group members are provided an equal opportunity to propose a project analysis plan. Analysis plans are developed and submitted in a standardized format with the help of the Working Group Chairs and then presented to the full Working Group membership via email and conference calls. Project leaders pitch their analysis plans to the Working Group and each member reviews and decides whether to contribute their data sample to the proposed projects (opt‐in). Most analysis plans earn widespread participation from the BD Working Group members, resulting in large‐scale studies that could not have been accomplished independently.

The BD Working Group's initial goal was to demonstrate the power and feasibility of large‐scale, collaborative neuroimaging analyses by establishing minimal bureaucratic barriers for participation and using standardized and publicly available processing and analysis protocols **(**Figures 2 and 3 **)**. To date, the BD Working Group has published five peer‐reviewed studies and currently coordinates over 18 active and ongoing analysis projects led by Working Group members from around the world.

## 3 f PUBLISHED STUDIES

### 3.1 Overview

The ENIGMA Bipolar Disorder Working Group has published five peer‐reviewed studies, each representing the largest neuroimaging studies of their kind and helping to answer the question of which brain structures are reliably associated with BD, its subtypes, and other clinical measures such as illness duration, severity, genetic risk, and common medications. Overall, these studies point to a diffuse pattern of brain alterations including smaller subcortical volumes, lower cortical thickness and altered white matter integrity in groups of individuals with BD compared to healthy controls. Small to moderate effect sizes are observed for ENIGMA‐standardized brain measures, in line with prior reports from the BD literature. Common medications such as lithium appear to have a normalizing effect on gray and white matter structures, whereas other treatments such as anticonvulsants appear to have the opposite effect. Standardized, ROI‐based cortical and subcortical brain measures were useful in parsing differences between patients and non‐affected relatives as well as providing above chance predictive accuracy in classifying BD individuals from controls using common machine learning techniques.

### 3.2 Alterations to subcortical volumes and associations with common pharmacological treatments

**FIGURE 4:**
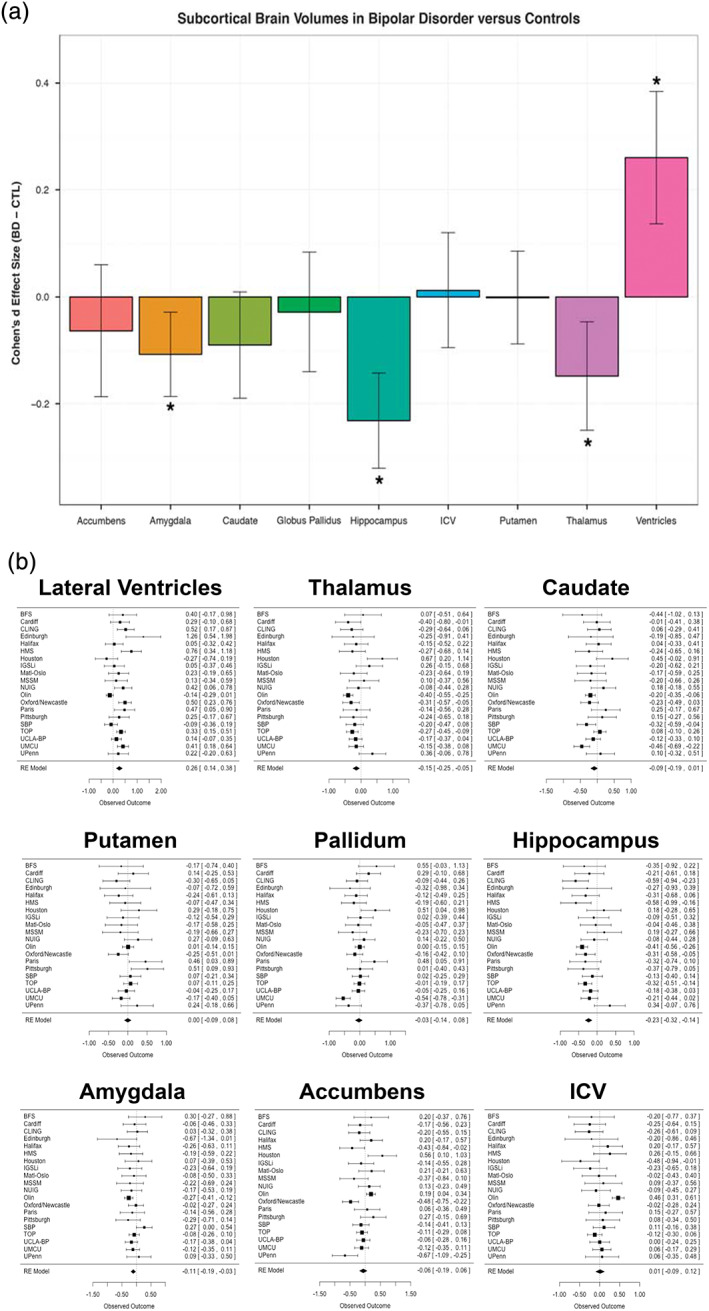
Findings from *Subcortical volumetric abnormalities in bipolar disorder* (Hibar et al., 2016). (a) Cohen's *d* effect size estimates for subcortical differences between individuals with BD versus healthy controls (HC) using ENIGMA‐standardized FreeSurfer volumes. Statistical model accounts for age, sex, and intracranial volume. Error bars indicate mean effect size ± standard error of the mean. Results passing study‐wide significance threshold are indicated by (*) including the amygdala which showed a trending effect. (b) Forest plots displaying the effect size estimates (adjusted Cohen's *d*) for each of the 20 study sites in the comparison of individuals with BD versus HC at each subcortical structure along with the overall inverse variance‐weighted random‐effects meta‐analysis results (RE Model)

The ENIGMA BD Working Group's first study was a meta‐analysis of subcortical gray matter brain volumes in 1,710 BD participants and 2,594 HC from 20 international sites, to identify effects of the disorder on regional morphometry, and rank structural brain metrics for case–control differences. On average, higher bilateral ventricular volumes and lower hippocampal, amygdala and thalamic volumes were detected in BD versus HC **(**Figure 4a **)** (Hibar et al., 2016). Importantly, all case–control effect sizes were small to moderate and varied to some degree across the 20 study samples. When combining the effects across sites meta‐analytically, clearer patterns emerged (Figure 4b). The group differences may reflect either accelerated atrophy in BD with potential disease‐related neuroprogression, chronic effects of the illness, or medication. Alternatively, smaller volumes may represent a potential risk factor for BD arising in early stages of development. Importantly, prior meta‐analyses were either unable to detect case–control differences in amygdala volume or reported variable effects (Altshuler et al., 2000; Chang et al., 2005).

No structural brain differences were detected between BD subtypes (BD‐I, BD‐II and BD‐NOS). Lithium treatment was associated with larger thalamic volumes compared to non‐treated individuals with BD. Compared to HC, individuals with BD not on lithium treatment had smaller hippocampal and thalamic volumes, on average, and larger lateral ventricles. Participants with BD taking anticonvulsants had smaller hippocampal volumes compared to non‐treated participants with BD. As discussed in the published study, these cross‐sectional results should be interpreted cautiously as medication status is confounded to some extent with illness characteristics (such as symptom severity). Furthermore, a simple binary coding (prescribed/not prescribed) was used to determine medication status at the time of scan. Current studies are investigating such medication factors as treatment history, dose, and serum level, to better model interactions between different pharmacological agents – polypharmacy is common in BD – and their associated effects on brain structure (see Ongoing and Future Studies section below and Supplemental Materials).

### 3.3 Widespread cortical thickness alterations in BD and associations with pharmacological treatment

**FIGURE 5:**
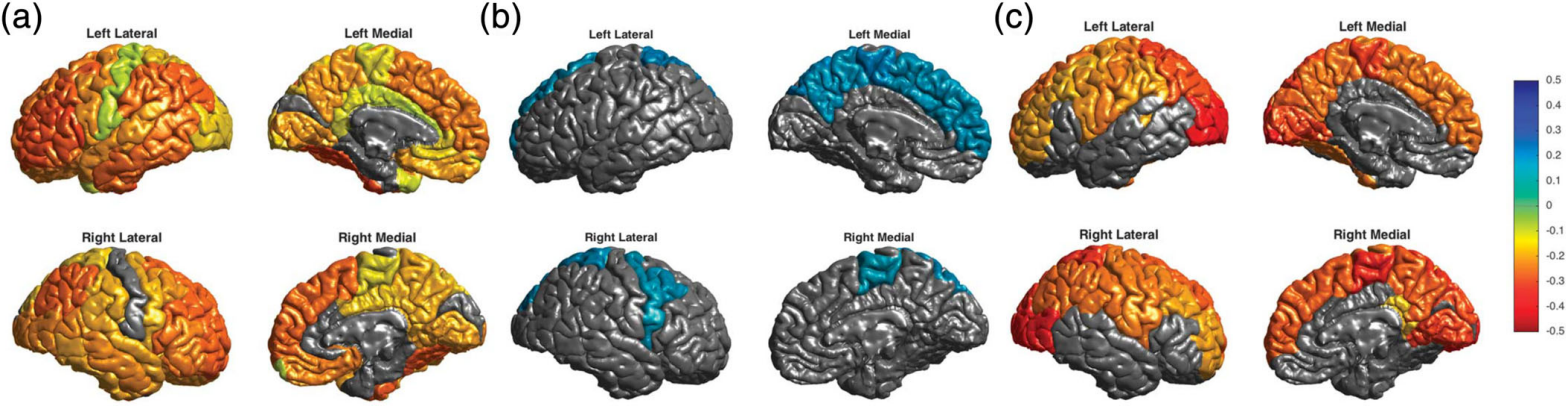
Findings from *Cortical abnormalities in bipolar disorder: an MRI analysis of 6,503 individuals from the ENIGMA Bipolar Disorder Working Group* (Hibar et al., 2018). (a) A widespread pattern of thinner cortex in adult individuals with BD versus HC. Cohen's *d* effect sizes plotted in regions passing correction for multiple comparisons. (b) Thicker cortex in adult individuals with BD taking lithium medication at time of scan. (c) Thinner cortex in adult individuals with BD associated with anticonvulsant treatment at time of scan

Prior meta‐analyses reported lower cortical thickness in the anterior cingulate, paracingulate, superior temporal gyrus and prefrontal regions associated with BD (Hanford, Nazarov, Hall, & Sassi, 2016; Phillips & Swartz, 2014). Surface area findings have been more variable **–** some larger studies detected no differences between BD and HC **(**Rimol et al., 2010 **)**. In our second study, again the largest of its kind, we focused on cortical structure (2,447 BD and 4,056 HC), examining ENIGMA‐standardized measures of cortical thickness and surface area in an expanded ENIGMA BD sample including 28 international sites (Hibar et al., 2018). Compared to controls, individuals with BD exhibited a widespread pattern of thinner cortex **(**Figure 5a **)**. Interestingly, and in agreement with previous large sample studies, no case–control differences were detected for cortical surface area. Longer illness duration was associated with a pattern of lower cortical thickness but not with surface area alterations. As in the subcortical study, no significant differences were detected between BD clinical subtypes.

With regard to medication status, we found significantly higher cortical thickness in participants with BD also taking lithium at the time of scan, with the largest effects in the left paracentral gyrus **(**Figure 5b **)**. Anticonvulsant treatment was associated with lower cortical thickness, with the greatest effects in bilateral occipital gyri **(**Figure 5C **)**. Atypical “second‐generation” antipsychotics were associated with lower cortical surface area in the rostral middle frontal gyrus, whereas typical “first‐generation” antipsychotics were associated with higher surface area in the left inferior parietal gyrus.

The cortical findings were largely in line with prior reports of thinner frontal and temporal cortices in BD. Notably, regions with the largest BD versus HC differences included the ventrolateral prefrontal cortex, an area long implicated in BD pathophysiology. Important new contributions include the observation of lower thickness in inferior parietal, fusiform, and inferior temporal regions in adults with BD. Structural deficits in these regions have been tied to disruptions in sensorimotor integration (Caspers, Zilles, Laird, & Eickhoff, 2010) and language (Vigneau et al., 2006), and may relate to altered emotion perception and rapid mood changes in BD. Further studies are needed to probe the functional relevance of regional cortical differences, and how these effects may be interrelated (e.g., using structural covariance analysis).

### 3.4 Multi‐site machine learning using brain MRI to identify bipolar disorder

**FIGURE 6:**
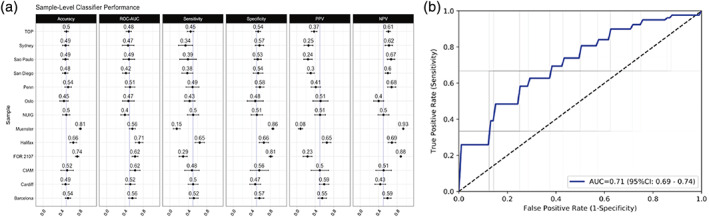
Findings from *Using structural MRI to identify bipolar disorders ‐ 13 site machine learning study in 3020 individuals from the ENIGMA Bipolar Disorders Working Group* (Nunes et al., 2018). (a) Support vector machine (SVM) classifier performance trained on each site independently, including mean and 95% confidence intervals for accuracy, area under the receiver operating curve (ROC‐AUC), sensitivity, specificity, positive predictive value (PPV) and negative predictive value (NPV). (b) Receiver operating curves from aggregate individual‐level analysis with dashed line indicating chance performance, blue line indicating mean ROC and gray lines indicating ROC curves from individual folds

Differential diagnosis of BD remains a challenge, with mis‐diagnosis leading to delays in effective treatments. In an effort to improve diagnostic accuracy and, ultimately, personalize treatments, machine learning can be used to find complex patterns in neuroimaging data that predict diagnostic categories, or prognosis. In our first such study, the Bipolar Working Group evaluated the capacity of a linear support vector machine classifier to predict the diagnosis of BD using standardized cortical and subcortical ROI‐based brain features from 853 individuals with BD and 2,167 HC acquired from 13 international sites (Nunes et al., 2018). Under appropriate cross‐validation procedures, diagnosis of BD was classified with a sensitivity of 0.66 (95% CI [0.63, 0.69]), a specificity of 0.65 (0.62, 0.67) and an area under the receiver operating characteristic (ROC) curve of 0.71 (0.69, 0.74) **(**Figure 6 **)**. Informative features were in agreement with previous findings, including the importance of hippocampus, amygdala (Hajek et al., 2009, 2012), and cortical regions such as inferior frontal and precentral gyri (Hibar et al., 2018).

When interpreting these results, we considered several issues. BD and its clinical subtypes are difficult to diagnose leading to noise in the target labels (i.e., derived brain measures). In addition, the illness shows marked clinical and neurobiological heterogeneity. Many brain alterations in BD are likely secondary to illness burden, presence or absence of comorbid conditions (Hajek et al., 2014; Hajek, McIntyre, & Alda, 2016), or treatments (Hibar et al., 2018; Van Gestel et al., 2019). Since these secondary changes are not found in all participants and reflect factors beyond the diagnosis, they cannot be used diagnostically. Last but not least, we worked with regional brain measures, not raw/voxelwise data. This approach involves information loss in the feature engineering process and using raw data could improve classification accuracy. While clinician judgment for BD diagnosis and treatment continues to outperform robust machine learning methods such as those studied here, the accuracy observed in this study provides a realistic and fair estimate of classification performance, which can be achieved in a large, ecologically valid, multisite sample of individuals with BD based on regional brain structure measures.

Our study provided further clues about the impact of data handling on classification performance. Performing the machine learning analyses on data pooled across the sites yielded a much better performance than meta‐analysis of site level results – the typical analytic method in multisite collaborations. Thus, future multisite brain‐imaging machine learning studies should attempt to move toward sharing of individual, raw data, not only site‐level results. Developing an ethico‐legal framework to facilitate safe sharing of raw data is a key and critical component of advancing medical machine learning (Passos et al., 2019).

### 3.5 Diffuse white matter alterations challenging the existing models of bipolar disorder

**FIGURE 7:**
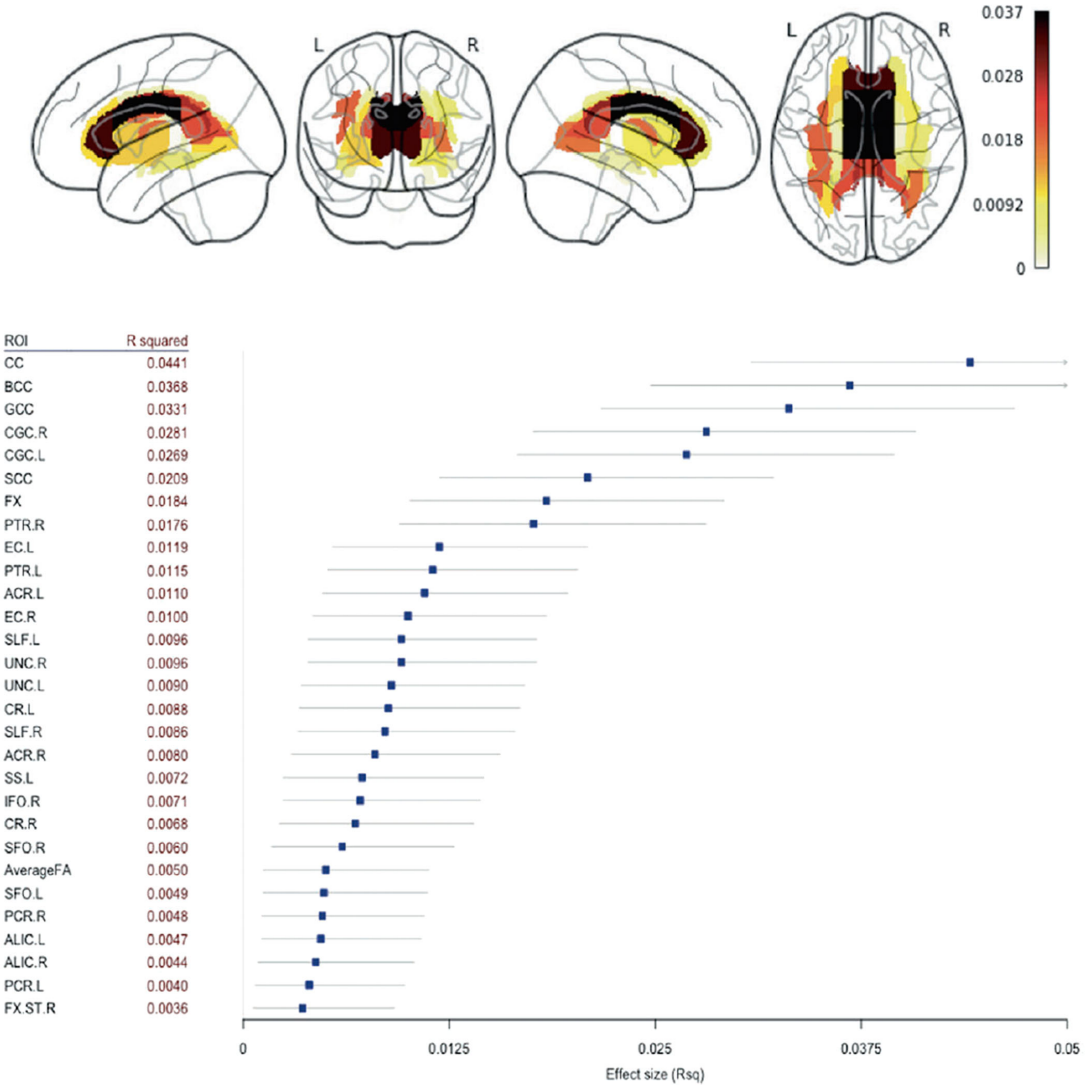
Findings from *Widespread white matter microstructural abnormalities in bipolar disorder: evidence from mega‐ and meta‐analyses across 3,033 individuals* (Favre et al., 2019). Mega‐analysis fractional anisotropy (FA) differences between BD and HC across 43 white matter (WM) tracts and the whole‐brain skeleton with R squared effect sizes and confidence intervals ranked by increasing order of magnitude for the regions showing significant group differences. R, right; .L, left; CC, corpus callosum; BCC, body of the corpus callosum; GCC, genu of the corpus callosum; CGC, cingulum; SCC, splenium of corpus callosum; FX, fornix; PTR, posterior thalamic radiation; EC, external capsule; ACR, anterior corona radiata; SLF, superior longitudinal fasciculus; UNC, uncinate fasciculus; CR, corona radiata; SS, sagittal stratum; IFO, inferior fronto‐occipital fasciculus, SFO, superior fronto‐occipital fasciculus; Average FA, average FA across full skeleton; PCR, posterior corona radiata; ALIC, anterior limb of the internal capsule; FXST, fornix (cres) / stria terminalis

Diffusion tensor imaging (DTI) studies of BD implicate widespread white matter (WM) alterations within and beyond the fronto‐limbic regions that appear to precede emotional instability (Phillips & Swartz, 2014). Limbic and non‐limbic tract disruptions have been reported (Canales‐Rodriguez et al., 2014) but inconsistencies exist in the literature and are likely due to the aforementioned research challenges. In our first DTI project, we aimed to identify generalizable WM microstructural alterations with a standardized processing and analysis framework (Jahanshad et al., 2013; Smith et al., 2006) to study case–control differences, as well as associations with clinical characteristics in BD. The project pooled data from 1,482 individuals with BD and 1,551 HC from 26 research samples across 12 countries. We used both meta‐ and mega‐analyses to form the largest multicenter DTI study of BD to date. Fractional anisotropy (FA) – a measure of white matter directionality, coherence and integrity – was lower, on average, in participants with BD across 29 out of 44 WM regions of interest, with strongest effects in the corpus callosum and cingulum **(**Figure 7 **)**. Higher FA was associated with later disorder onset and shorter illness duration. Lithium treatment was associated with higher FA, both globally and in a number of regions of interest including the corona radiata, posterior thalamic radiation and internal capsule (Favre et al., 2019). No significant FA alterations were detected between BD subtypes (BD‐I and BD‐II diagnosis) or associated with antidepressant use, illness severity (measured by number of mood episodes/duration of illness) or history of psychotic symptoms.

Unlike prior studies, we reported widespread FA alterations in BD **–** a pattern similar to results from the ENIGMA Schizophrenia Working Group DTI study (Kelly et al., 2018). This suggests that a global pattern of microstructural abnormalities may span both disorders. Altered WM microstructure in the cingulum, a major limbic pathway, is in agreement with prior reports of disrupted fronto‐limbic connectivity in BD (Mahon, Burdick, & Szeszko, 2010; Phillips & Swartz, 2014). However, the role of the corpus callosum in BD is not clear. WM alterations in individuals with BD with psychotic symptoms have been reported (Sarrazin et al., 2014), but the role of the corpus callosum in emotion processing or mood switching is not fully understood (Linke et al., 2013; Wang et al., 2008). Lower corpus callosum FA was reported in the ENIGMA DTI meta‐analysis of SCZ (Kelly et al., 2018) and MDD (van Velzen et al., 2019), suggesting overlapping pathophysiology in psychosis and affective disorders (Koshiyama et al., 2019). Further studies are needed to evaluate the extent to which the corpus callosum might be differentially affected in these related disorders. Preliminary data suggest that disruption of interhemispheric connectivity is a disease marker rather than a vulnerability marker to BD (Chepenik et al., 2010; Linke et al., 2013). Nonetheless, we identified extensive FA‐related WM abnormalities, which challenges current pathophysiological models of BD. Future models should not be limited to fronto‐limbic networks, and should consider interhemispheric dysconnectivity as a feature of interest in BD.

An important limitations of the study was the focus on the most commonly used DTI measure, FA, as opposed to including other scalar measures such as mean, axial, and radial diffusivity derived from the tensor model. The tensor, the most common method of modeling diffusion MRI, while robust and widely used, has well documented limitations such as inability to resolve crossing fibers – which are present in a large proportion of brain white matter – or to disentangle the intra‐ and extra‐axonal compartments. As detailed below, ongoing studies from the ENIGMA BD Working Group are currently applying more advanced analysis techniques to model white matter. Furthermore, future BD research will likely benefit from new diffusion MRI methods to model white matter tissue microstructure such as Neurite Orientation Dispersion and Density Imaging (NODDI) (Zhang, Schneider, Wheeler‐Kingshott, & Alexander, 2012), which has been used to highlight the impact of lithium on neurite density (Sarrazin et al., 2019).

### 3.6 Mapping familial risk to brain structure across bipolar disorder and schizophrenia

Most neuroimaging studies to date have compared individuals with BD to HC. The interpretation of case–control findings is complicated by many of the aforementioned study limitations. An alternative approach is to study first‐degree family members (i.e., offspring, siblings, parents or co‐twins), as they are at higher risk for the disorder but are otherwise healthy and unmedicated. As first‐degree relatives share, on average, half of the genes with their ill relative (except for monozygotic co‐twins who share all their genes), a family design provides a unique angle from which to study the effect of BD risk on brain structure and function.

The ENIGMA‐Relatives Working Group has taken a cross‐disorder approach to the study of BD and SCZ relatives, motivated by overlapping clinical symptoms, including delusions, hallucinations, mania, depression and anxiety, as well as shared genetic and epidemiological risk (Cross‐Disorder Group of the Psychiatric Genomics Consortium, 2013; Kempf, Hussain, & Potash, 2005; Pearlson, 2015). While BD and SCZ do show distinct symptom patterns and clinical course (Bora, 2015; Correll, Penzner, Frederickson, et al., 2007; Murray & Sham, 2004), it remains unclear whether they represent discrete entities shaped by distinct etiology and pathogenesis, or if they represent a spectrum of mood‐psychosis disorders. The ENIGMA‐Relatives Working Group has aimed to identify overlapping and distinct features of SZ and BD in first‐degree relatives of patients with these disorders.

**FIGURE 8:**
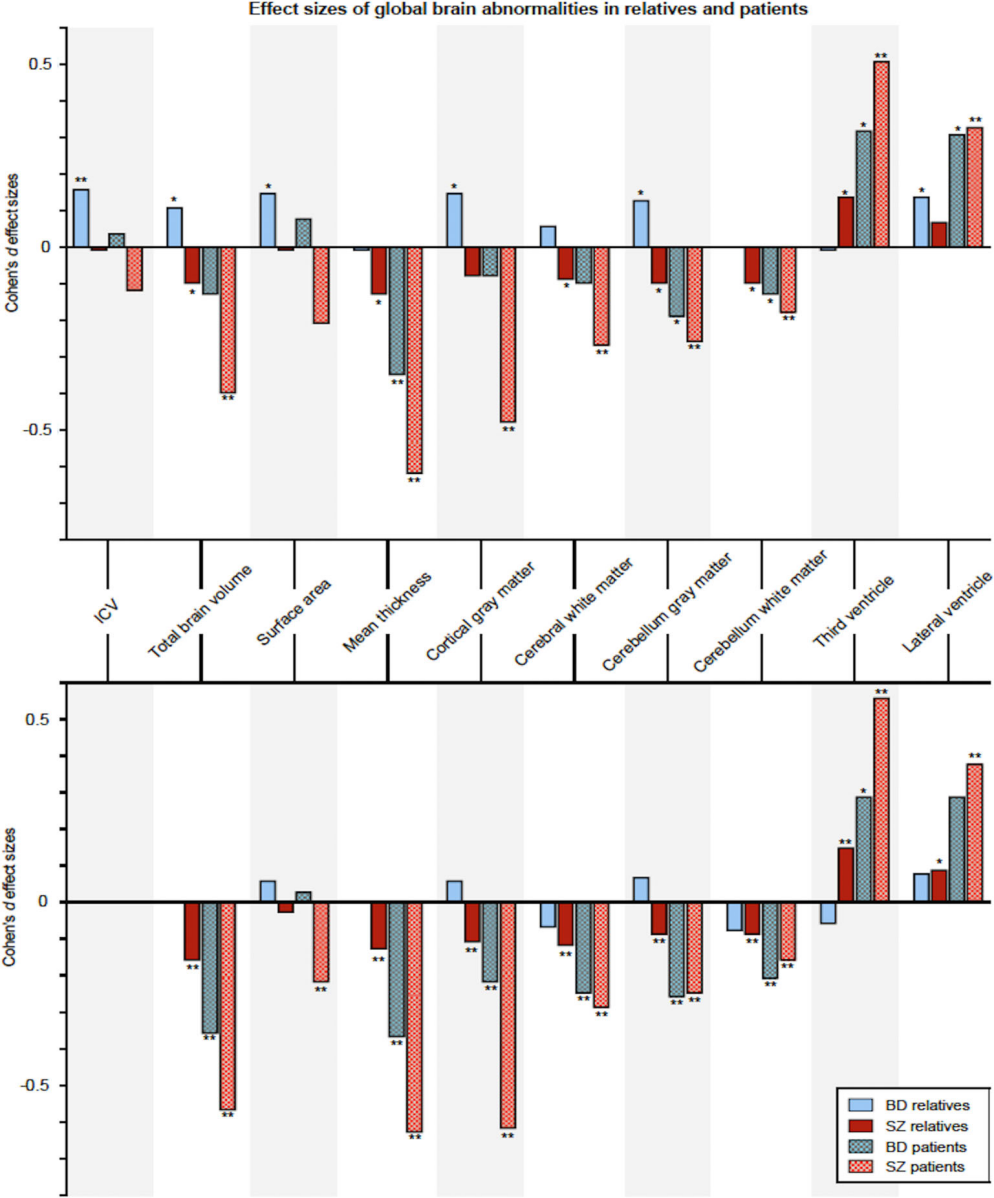
Findings from *The association between familial risk and brain abnormalities is disease‐specific: an ENIGMA–Relatives study of schizophrenia and bipolar disorder* (de Zwarte et al., 2019). Top: Cohen's *d* effect sizes comparing BD and SCZ relatives and healthy controls across global brain measures. Bottom: global effect sizes adjusted for total intracranial volume (ICV). *Nominally significant (*p* < .05 uncorrected); **q < .05 corrected for multiple comparisons

In the largest study to date, standardized subcortical and global brain measures were meta‐analyzed across healthy first‐degree relatives of individuals with either BD (FDRs‐BD) or SZ (FDRs‐SZ) (de Zwarte, Brouwer, Agartz, et al., 2019). A total of 6,008 participants from 34 family cohorts, including 1,228 FDRs‐SZ, 852 FDRs‐BD, 2,246 HC, 1,016 participants with SZ and 666 with BD were included. The main findings included: 1) FDRs‐BD had larger average intracranial volumes (ICV), whereas FDRs‐SZ showed smaller thalamic volumes compared with HC, 2) in FDRs‐BD, ICV explained the larger brain volumes in other regions, whereas in FDRs‐SZ, brain volumes and thickness effect sizes became significantly smaller compared to HC after statistical correction for ICV, 3) brain alterations differed between the relative types, but no clear pattern was detected, and 4) findings were not confounded by other psychiatric diagnoses in the relatives **(**Figure 8 **)**.

When considering the finding of larger ICV in FDRs‐BD but not FDRs‐SZ, as well as prior reports indicating a diverging pattern of ICV volume for individuals with BD and SCZ (i.e., significantly smaller ICV in SZ but not BD) (Hibar et al., 2016; van Erp et al., 2016), the ENIGMA‐Relatives Working Group recently investigated whether differences in Intelligence Quotient (IQ) or educational attainment could explain the findings in SZ and BD relatives. These most recent findings are detailed in de Zwarte et al. from this issue.

## 4 ONGOING AND FUTURE STUDIES

Building on these initial studies, the ENIGMA BD Working Group has a growing list of ongoing projects to: (a) more finely map the initial ROI‐based findings of altered cortical/subcortical gray matter and WM alterations in BD by using advanced, high‐definition brain morphometric features including vertex‐ or voxel‐wise analysis, (b) improve classification and individual‐level prediction using data driven clustering/biotyping with more advanced machine learning techniques, (c) investigate the impact of polygenic risk and gene expression markers on large‐scale standardized brain measures, (d) empower future mega‐analyses that allow more sophisticated designs than meta‐analyses, and (e) study how individual symptoms, functional domains and risk factors map onto measures of brain structure and function that cross classic diagnostic boundaries, revealing shared and distinct brain markers of mental illness. Further details of the ongoing Bipolar Disorder Working Group projects may be found in the Supplemental Materials.

### 4.1 Finer mapping of BD‐related brain variation

The ENIGMA Bipolar Working Group is currently applying advanced techniques that model subcortical shape morphometry, hippocampal subfield volumes, WM connectivity, neurometabolites, longitudinal brain change and brain aging across thousands of individuals with BD and HC. These methods, now standardized for multisite studies, aim to better characterize the spatial distribution of BD‐related alterations across the brain, track the longitudinal trajectory of such brain changes across the course of illness, and determine the extent to which those changes may interact with normal brain aging processes.

### 4.2 Machine learning for better classification and individual‐level prediction

Most ENIGMA projects to date have applied relatively simple mass univariate analysis methods when studying brain features. Combining multiple imaging modalities will likely improve predictions of diagnosis and prognosis. In addition to classical multivariate approaches, machine learning models are being applied to neuroimaging data across a variety of ENIGMA BD projects to predict diagnostic groups, treatment response, and to characterize subtypes or clusters within groups of individuals diagnosed with BD. Our initial efforts in the diagnostic classification of BD showed promise (Nunes et al., 2018), but accuracy is likely to improve with the addition of functional neuroimaging measures (Han, De Berardis, Fornaro, & Kim, 2019; Phillips & Vieta, 2007), as well as techniques that merge multimodal structural, functional, vertex‐wise metrics and in‐depth clinical information. The BD Working Group is tackling new challenges that arise when using higher‐dimensional data and when fitting complex models to provide improved individual‐level predictions.

### 4.3 Large‐scale direct comparisons of brain measures across psychiatric disorders

A key question in psychiatric neuroimaging is the extent to which brain variations are shared or differentiate major psychiatric disorders. Many mental illnesses overlap in symptomatology, response to medication and underlying genetic risk. Examples include known intersections between BD and MDD and SCZ (Pearlson, 2015; Rink, Pagel, Franklin, & Baethge, 2016). Whether shared clinical features reflect similar underlying brain structure and function is poorly understood. As many clinically‐focused ENIGMA Working Groups have completed studies of cortical and subcortical structure, direct comparison of brain measures for tens of thousands of participants is now possible. The resulting data set represents the largest collection of psychiatric neuroimaging data ever amassed using standardized processing techniques, and the largest samples of BD, MDD, and SCZ neuroimaging data ever analyzed.

**FIGURE 9:**
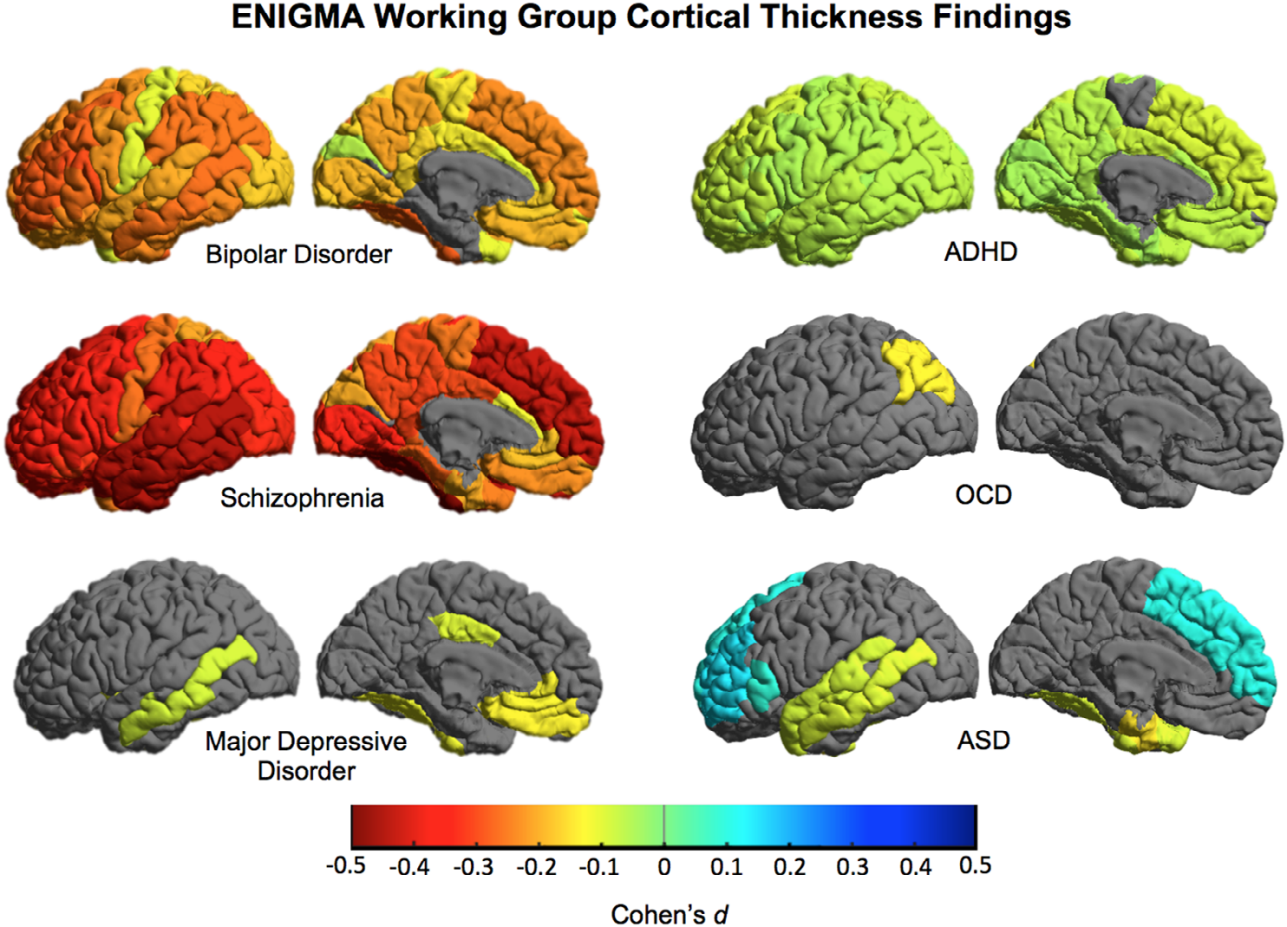
Cortical thickness differences across ENIGMA working groups. Cohen's *d* effect sizes comparing cases versus healthy controls (HC) plotted across 34 bilateral cortical ROIs from ENIGMA‐standardized FreeSurfer protocol (http://enigma.ini.usc.edu/protocols/). Warmer colors indicate lower thickness in cases/patients, whereas cooler colors indicate greater thickness in cases/patients versus HC. Results derived from published ENIGMA studies: bipolar disorder (*N* = 4,419, 28 sites, Hibar et al., 2018), major depressive disorder (*N* = 10,105, 15 sites, Schmaal et al., 2017), schizophrenia (*N* = 9,572, 39 sites, van Erp et al., 2018), attention deficit hyperactivity disorder (ADHD *N* = 4,180, 36 sites, Hoogman et al., 2019), obsessive–compulsive disorder (OCD *N* = 3,665, 27 sites, Boedhoe et al., 2018) and autism spectrum disorder (ASD *N* = 3,222, 49 sites, van Rooij et al., 2018)

The initial case–control studies from the ENIGMA clinical working groups **(**Figure 9 **)** suggest overlapping and distinct patterns of brain alterations across disorders. Notably, ENIGMA SCZ case–control cortical effects were more widespread and greater in magnitude than those found in BD and MDD. In line with prior hypotheses regarding underlying biological correlates of BD versus MDD, frontal lobe systems showed greater deficits in ENIGMA BD cases, whereas limbic regions tended to show greater deficits in ENIGMA MDD cases. Smaller hippocampal volume is another common finding across published ENIGMA psychiatric studies. Preliminary analyses, correlating effect sizes across published ENIGMA psychiatric working group studies, indicate significant correlations between cortical and subcortical MRI alterations across disorders, which may be partially explained by common underlying genetic markers (Ching et al., 2020).

For pragmatic reasons, and to help incorporate the greatest number of Working Group sites in initial analyses, the first ENIGMA BD Working Group studies tended to include a limited number of essential variables such as age, sex, diagnosis, age of illness onset, binary (yes/no) medication status at the time of scan, and simple severity measures. The next phase of ENIGMA cross‐disorder analyses is tackling the challenges of deeper phenotyping and the heterogeneity across samples. The ENIGMA BD Working Group is collecting in‐depth clinical and demographic information in a harmonized effort with the ENIGMA SCZ and MDD Working Groups from over 200 data sets. Metrics of interest include education, socioeconomic status, IQ, body mass index, number of psychiatric hospitalizations, number of episodes (depressive, manic, psychotic), substance use disorder, comorbid psychiatric disorders, current/lifetime medication treatment, medication dose, medication serum level, and detailed behavioral/symptom information. These measures will provide the basis for comparisons not dependent on classic diagnostic categorizations. Work is underway to find overlapping scales and measures and to resolve clinical/behavioral measurement harmonization and empower analyses more in line with the Research Domain Criteria (RDoC) framework (Insel, 2014). Clinical information will also allow for analyses such as network modeling of individual symptoms (Borsboom & Cramer, 2013) and thus a more detailed characterization than traditional diagnostic categories. Such modeling can also be done in combination with brain and other biological measures, as in recent studies of MDD (Fried et al., 2019; Hilland et al., 2019).

Advanced supervised and unsupervised machine learning techniques are being applied to examine the extent to which BD, MDD and SCZ can be discriminated on the basis of ENIGMA‐standardized brain measures. Of particular interest is whether multivariate, machine learning techniques can discriminate between alternative patient groupings and cluster individuals according to duration of illness, symptom severity, presence of depressive and/or psychotic symptoms, substance use, treatment response and other characteristics that might improve individual‐level predictive accuracy.

### 4.4 Linking genetic risk and gene expression to brain alterations across psychiatric disorders

The ENIGMA bipolar disorder working group is uniquely positioned to study how genetic risk loci affect brain structure and function. In collaboration with the Psychiatric Genomics Consortium Working Group on Bipolar Disorders (PGC‐BD), we are deriving polygenic risk scores (PRS) across participating ENIGMA BD Working Group sites using recent discoveries from the PGC‐BD group (Bipolar Disorder and Schizophrenia Working Group of the PGC, 2018; Stahl et al., 2019). The ENIGMA PRS protocol is freely available online (http://enigma.ini.usc.edu/protocols/) and is empowering projects to map BD‐PRS vulnerability across brain structures, as well as genetic risk for other psychiatric disorders. This effort aims to both identify brain regions at risk in BD as well as construct overlapping genotype–phenotype risk maps across common psychiatric disorders, which may reveal more mechanistic models of shared and unique disease processes.

A key challenge in neuroimaging is bridging the gap between in vivo MRI and ex vivo histology (Paus, 2018). To address this, several ENIGMA working groups applied a “virtual histology” approach, where profiles of structural brain metrics, namely group differences in cortical thickness, are related to gradients in cell‐specific gene expression using data from the Allen Human Brain Atlas (Hawrylycz et al., 2012; Patel et al., 2018; Shin et al., 2017). Inter‐regional profiles of group differences in cortical thickness have been generated meta‐analytically across over 12,000 cases and 15,000 HC from the ENIGMA BD, ASD, ADHD, OCD, MDD and SCZ Working Groups. Associations of a given cell type with a profile of group differences in cortical thickness are correlated with cell‐specific gene expression to better characterize shared and unique gene expression potentially driving cortical alterations across these major neuropsychiatric disorders.

## 5 FUTURE PERSPECTIVES: REPLICATION, BIG DATA AND SHIFTING THE PARADIGM IN PSYCHIATRIC RESEARCH

BD is a complex illness where individual factors confer a small proportion of the overall risk. The ENIGMA Bipolar Disorder Working Group is focused on applying innovative, big data approaches to provide reliable new discoveries on the biological underpinnings of BD, and to generate clinically relevant findings to improve diagnosis and treatment.

The current psychiatric research paradigm, which seeks markers that link high‐level, behavioral‐based diagnostic labels to underlying biology, is slowly changing. Neuroimaging may not provide the larger BD‐related effect sizes once proposed by the “endophenotypes” hypothesis, but the combination of large‐scale neuroimaging data sets has led to a better understanding of the brain regions that mediate the link between genetic risk and the behavioral manifestations of BD.

Several limitations to the ENIGMA Bipolar Disorder Working Group approach must be discussed. First, Working Group sites collected data independently and used a range of tools and methods that can differ with respect to MRI scanner (hardware and software), biospecimen collection, behavioral assessment, study inclusion/exclusion criteria and overall data quality. While ENIGMA studies deploy standardized processing and analysis techniques that reduce the variance across site measurements, true data harmonization is only possible through prospective data collection. Second, the ENIGMA Bipolar Working Group consists of fairly Eurocentric data samples. Future efforts must be made to incorporate additional data sets from all corners of the world to garner an even more ecologically valid sample of BD phenotypes. Third, early ENIGMA studies initially deploy pragmatic analysis plans, using only the most common measures across Working Group sites (e.g., binary medication status at time of scan, primary diagnosis, etc.) and applying mass univariate analysis methods (e.g., BD case vs. HC differences assessed by multiple linear regression). While this approach has led to the incorporation of many standardized data sets and has helped to clarify basic brain structure alterations in BD, group‐level results only allow for broad generalizations about individuals with BD and have limited clinical applications when assessing and treating individual patients. Fourth, all of the published studies from the Bipolar Disorder Working Group have so far used a cross‐sectional design. There is increasing evidence that BD is a neuroprogressive disorder – our own results indicate associations between duration of illness and brain structure (Favre et al., 2019; Hibar et al., 2018) – which requires a longitudinal design that may inform better clinical staging and treatment of the illness (Salagre et al., 2018). Longitudinal studies that include participants with varying ages of onset may also help to address whether brain alterations are indeed inherent markers of BD or are a consequence of factors associated with illness duration. Longitudinal studies in the context of therapeutic trials will also clarify the effect of interventions on the brain, although there are many challenges in integrating such data across sites.

With these limitations in mind, the ENIGMA Bipolar Disorder Working Group has several future research goals:

1. *Incorporate advanced and standardized multimodal measures*. Combining multimodal biological measures that encompass genetic, neurochemical, neuroimaging and behavioral factors will likely lead to more clinically relevant biomarkers for improved patient‐level predictions and a better understanding of BD pathophysiology. Innovative standardized protocols now being applied in the ENIGMA Bipolar Disorder Working Group include white matter connectivity, resting and task‐based fMRI, spectroscopy, vertex‐wise shape morphometry, longitudinal brain change and deeper clinical and behavioral phenotyping.
2. *Advance novel, big data techniques*. We are currently applying advanced machine learning models to predict classic diagnostic groups, as well as potential patient subgroup stratification along behavioral and treatment measures. New multivariate GWAS methods are helping to reveal more of the hidden genetic risk for BD (e.g., the MOSTest, see van der Meer et al., 2019) and are being applied through our collaboration with the PGC. These genetic findings further empower the ENIGMA Bipolar Working Group's ability to map genetic risk to brain structure and function.
3. *Cross‐disorder studies*. Large‐scale, transdiagnostic efforts such as those being carried out between the ENIGMA BD, MDD, and SCZ working groups will help us understand the common and unique neurobiological factors underlying mental illnesses.
4. *Hypothesis‐driven prospective analyses*. Our international collaborations are driving future prospective studies with more harmonized data collection. Lessons learned from ongoing gross neuroimaging analyses inform future micro‐ and ultrascale studies of cellular morphometry, distribution, and synaptic structure and function, which may provide a more mechanistic link between genes and behavior in BD (Le & Stein, 2019). A centrally coordinated, long‐term study of a large cohort of individuals with BD across the lifespan is desperately needed. Such a study, collecting neuroimaging, cellular, molecular and other deeper phenotyping measures akin to studies such as UK Biobank and other longitudinal cohorts (McInnis & Greden, 2016; Miller et al., 2016; Yatham, Kauer‐Sant'Anna, Bond, Lam, & Torres, 2009), and integrated with pharmacological and behavioral treatment interventions would provide an ambitious path forward to addressing many of the key challenges facing BD research.

The ENIGMA BD Working Group has demonstrated that large‐scale, international collaborations can empower replicable and generalizable studies of BD. To the goal of more open and reproducible science, the ENIGMA Consortium is building an Organic Data Science (ODS) tool to facilitate complex and dynamic working group activities via a systematic information system. Based on Semantic MediaWiki, ENIGMA‐ODS provides cross‐working group data queries and project tracking to improve study reproducibility and help to overcome barriers to efficiency that are inherent in large‐scale projects (Jahanshad et al., 2015). Furthermore, the use of publicly available and standardized processing and analysis protocols may empower future “living studies” or continuously updated research findings (e.g., associations with subcortical volume and psychotic symptoms), with semi‐automated addition of future cohorts to an ever‐increasing ENIGMA‐standardized research sample.

Engaging patients is vital to ensure that biomedical research, and the subsequent interventions and tools, meet the needs of individuals living with BD. The Milken Institute and the Depression and Bipolar Support Alliance recently surveyed over 6,000 individuals living with MDD and BD to better understand research priorities from individuals living with these illnesses (Altimus, 2019). Survey respondents identified the ability to be independent or act according to one's own will as the top wellness priority, while only 20% of individuals identified the lack of symptoms of acute MDD or BD as a measure of wellness. Furthermore, 54% of respondents reported experiencing both MDD and BD symptoms, contrary to a discrete diagnosis given by clinicians. These discrepancies in both wellness priorities and manifestation of the disorder demonstrate that the most pressing needs of individuals with BD may not align with the goals of researchers. Moving forward, the ENIGMA BD working group aims to actively engage user groups to help focus our research goals and engage new participants in future studies.

The ENIGMA BD Working Group is actively recruiting new research collaborators and the infrastructure allows for new groups to be efficiently incorporated into ongoing and future analyses. Groups interested in joining, contributing data, and starting their own research proposal using the largest neuroimaging data set in BD research are encouraged to contact the Working Group Chairs.

The ENIGMA Bipolar Working Group will continue to be guided by the collective expertise of a strong network of neuroscientists, psychiatrists, data scientists, bioengineers and geneticists (Guglielmi, 2018). Big data consortia efforts offer the opportunity to work cohesively on related research questions, bringing diverse information to bear on neuroscientific problems, and will continue to provide valuable discoveries, revealing consensus findings and informing future hypothesis‐driven studies of BD and other neuropsychiatric disorders.

All images taken from original publications are approved for reprint under creative commons licensing.

## CONFLICT OF INTEREST

O. A. A. received Speaker's honorarium from Lundbeck and is a consultant for HealthLytix. M. B. was supported by an unrestricted grant from AstraZeneca. A. C. B. is a full‐time employee of P1vital Ltd. C. R. K. C. and P. M. T. have received partial research support from Biogen, Inc. (Boston, USA) for work unrelated to the topic of this manuscript. T. E. has received a speaker's fee from Lundbeck. G. M. G. is a NIHR Emeritus Senior Investigator, holds shares in P1vital and P1Vital products and has served as consultant, advisor or C. M. E. speaker in the last 3 years for Allergan, Angelini, Compass pathways, MSD, Janssen, Lundbeck (/Otsuka or /Takeda), Medscape, Minerva, P1Vital, Pfizer, Sage, Servier, Shire, Sun Pharma. D. P. H. is a full‐time employee of Genentech, Inc. A. M. M. has received research support from the Eli Lilly, Janssen and The Sackler Trust. J. C. S. has participated in research funded by Forest, Merck, BMS, and GSK and has been a speaker for Pfizer and Abbott. Marsal Sanches has received research grants from Janssen. All other authors from this site report no conflicts of interest to declare. D. J. S. has received research grants and/or consultancy honoraria from Lundbeck and Sun. E. V. has received grants and served as consultant, advisor or CME speaker for the following entities (work unrelated to the topic of this manuscript): AB‐Biotics, Abbott, Allergan, Angelini, Dainippon Sumitomo Pharma, Galenica, Janssen, Lundbeck, Novartis, Otsuka, Sage, Sanofi‐Aventis, and Takeda.

## Supporting information


**Appendix S1**: Supporting Information

## Data Availability

This is a review article of the ENIGMA Bipolar Disorder Working Group activities and includes no primary data. Those that wish to become members of the ENIGMA Bipolar Disorder Working Group are encouraged to contact the Working Group Chairs.

## ENDNOTE

^1^
A biomarker is an objective genetic, biomolecular or morphological indicator of a normal/pathological process or biological response to therapeutic intervention used for diagnosis or prognosis (Atkinson et al., 2001; Venkatasubramanian & Keshavan, 2016).

## APPENDIX A MEMBERS OF ENIGMA‐BIPOLAR DISORDER WORKING GROUP

Geraldo F. Busatto (Laboratory of Psychiatric Neuroimaging (LIM‐21), Departamento e Instituto de Psiquiatria, Hospital das Clinicas HCFMUSP, Faculdade de Medicina, Universidade de São Paulo, São Paulo, SP, Brazil)

Tiffany M. Chaim‐Avancini (Laboratory of Psychiatric Neuroimaging (LIM‐21), Departamento e Instituto de Psiquiatria, Hospital das Clinicas HCFMUSP, Faculdade de Medicina, Universidade de São Paulo, São Paulo, SP, Brazil)

Sara Dallaspezia (Vita‐Salute San Raffaele University, Milan, Italy; Division of Neuroscience, Psychiatry and Psychobiology Unit, IRCCS San Raffaele Scientific Institute, Milan, Italy)

Benjamin I. Goldstein (Centre for Youth Bipolar Disorder, Sunnybrook, Toronto, Ontario, Canada)

Guy M. Goodwin (Department of Psychiatry, University of Oxford, Oxford, England)

Pedro G. P. Rosa (Laboratory of Psychiatric Neuroimaging (LIM‐21), Departamento e Instituto de Psiquiatria, Hospital das Clinicas HCFMUSP, Faculdade de Medicina, Universidade de São Paulo, SP, Brazil)

Naoki Hashimoto (Department of Psychiatry, Hokkaido University Graduate School of Medicine, Sapporo, Japan)

Matthew J. Kempton (Institute of Psychiatry, Psychology & Neuroscience, King's College London, London, England)

Axel Krug (Department of Psychiatry and Psychotherapy, Philipps‐University Marburg, Marburg, Germany; Department of Psychiatry and Psychtherapy, University of Bonn, Germany)

Hiroshi Kunugi (Department of Mental Disorder Research, National Institute of Neuroscience, National Center of Neurology and Psychiatry, Tokyo, Japan)

Rayus Kuplicki (Laureate Institute for Brain Research, Tulsa, Oklahoma)

Clare E. Mackay (Department of Psychiatry, University of Oxford, Oxford, England)

Urlik F. Malt (Institute of Clinical Medicine, University of Oslo, Oslo, Norway)

Koji Matsuo (Department of Psychiatry, Faculty of Medicine, Saitama Medical University, Moroyama, Iruma, Japan)

Andrew M. McIntosh (Centre for Clinical Brain Sciences, University of Edinburgh, Edinburgh, England)

Sean R. McWhinney (Department of Psychiatry, Dalhousie University, Halifax, Nova Scotia, Canada)

Hisashi Narita (Department of Psychiatry, Hokkaido University Graduate School of Medicine, Sapporo, Japan)

Jonathan Savitz (Laureate Institute for Brain Research, Tulsa, Oklahoma, USA; Oxley College of Health Sciences, The University of Tulsa, Tulsa, Oklahoma)

Henk S. Temmingh (Department of Psychiatry and Mental Health, University of Cape Town, Cape Town, South Africa; Valkenberg Hospital, Western Cape Department of Health, Observatory, South Africa)

Sarah Trost (Department of Psychiatry and Psychotherapy, University Medical Center Goettingen, Goettingen, Germany)

Holly B. Van Gestel (Department of Psychiatry, Dalhousie University, Halifax, Nova Scotia, Canada)

Amelia Versace (University of Pittsburgh, Pittsburgh, Pennsylvania)

Marcus V. Zanetti (Laboratory of Psychiatric Neuroimaging (LIM‐21), Departamento e Instituto de Psiquiatria, Hospital das Clinicas HCFMUSP, Faculdade de Medicina, Universidade de São Paulo, São Paulo, SP, Brazil; Instituto de Ensino e Pesquisa, Hospital Sírio‐Libanês, São Paulo, SP, Brazil)

Laboratory of Psychiatric Neuroimaging (LIM‐21), Departamento e Instituto de Psiquiatria, Hospital das Clinicas HCFMUSP, Faculdade de Medicina, Universidade de São Paulo, São Paulo, SP, Brazil
