# Supplementary material for: What we learn about bipolar disorder from large‐scale neuroimaging: Findings and future directions from the ENIGMA Bipolar Disorder Working Group
Source: Hum Brain Mapp. 2020 Jul 29;43(1):56–82. doi: 10.1002/hbm.25098 (PMC8675426; doi:10.1002/hbm.25098)
Supplement: Supplementary file 1 — Appendix S1: Supporting Information [file HBM-43-56-s001.pdf]

Supplemental Materials

Major challenges in bipolar disorder research

On average, a 5-10 year gap still exists from the time of symptom onset to BD diagnosis (Berk et al., 2007). This delay may lead to greater illness severity, comorbidities, hospitalizations, suicide risk, and a detrimental impact on patients' education, employment and quality of life (Judd & Akiskal, 2003; Keck et al. 2008; Altamura et al., 2010). It is estimated that only 20% of patients with BD presenting with depression are properly diagnosed with BD in their first year seeking treatment (Goldberg et al., 2001). Individuals with BD have one of the highest suicide rates among psychiatric patients, with upwards of 50% of individuals with BD attempting suicide, and a rate of completed suicide ~20 times higher than the general population (Schaffer et al., 2015; Pompili et al., 2013; Grande et al., 2016).

Individuals with BD often present with comorbidities, including other common psychiatric disorders (e.g., substance use, attention deficit hyperactivity, anxiety and personality disorders), as well as non-psychiatric disorders (e.g., diabetes, fibromyalgia, osteoporosis, metabolic syndrome, cardiovascular disease, endocrine disorders). All of these comorbid conditions create additional challenges for diagnosis, treatment and prognosis (Vieta et al., 2018.). For example, most patients with BD present with depressive episodes that are often clinically indistinguishable from those in major depressive disorder (MDD). While the DSM-5 criteria for depressive episodes in BD and MDD are the same, the extent to which the underlying biological markers of depressive symptoms in each disorder overlap or are distinct remains unknown. A number of subtle clinical aspects may help to differentiate them from one another at a group-wise level, including age of onset, episode duration/frequency, phenomenology such as atypical features, response to treatment, and comorbid substance use (Grande et al., 2016; Goodwin et al., 2008), but further studies in large, well-powered samples are needed.

While many patients may achieve periods of remission, rates of recurrent mood episodes are high, even during treatment (Gitlin et al., 1995; Solomon et al., 1995;

Perlis et al., 2006; Vázquez et al., 2015), with long-term, subthreshold symptoms commonly impeding full recovery. About two thirds of individuals with BD have global or selective neurocognitive deficits, including impaired executive functioning and verbal memory even in periods of remission (Rosa et al., 2012; Bourne et al., 2013; Martinez-Aran & Vieta, 2015; Sparding et al., 2015, 2017), which can impact daily functioning (Depp et al., 2012). Although cognitive deficits are present even in the early stages after remission of the first manic episode (Torres et al., 2010), the magnitude of such deficits tends to be small. Further, cognitive deficits in early stages may be reversible if mood episodes can be prevented (Kozicky et al., 2014), suggesting early and optimal intervention as a critical goal in arresting potential BD neuroprogression. In BD patients with multiple mood episodes, although preliminary data supports the utility of cognitive/functional remediation (Lewandowski et al., 2017, Sanchez-Moreno et al., 2017) and 5-HT7 antagonists (Yatham et al., 2017) in improving cognitive functioning, further work is needed.

A key hurdle facing psychiatric research is the heavy reliance on linking categorical diagnostic labels to underlying biological measures (e.g., single nucleotide polymorphisms, plasma markers, neurotransmitters, regional brain volumes, etc.). While some progress has been made in associating current BD diagnostic constructs to biological markers, from genes to neurochemistry to brain structure to behavior, the current diagnostic labels, which discretely categorize groupings of complex and heterogeneous symptoms, remain difficult to map onto underlying pathophysiological mechanisms. For instance, based on DSM-5 criteria, there exist ~37,264 symptom combinations that would meet the minimal threshold for a BD-I diagnosis alone, not including BD-II or any specifier. Such heterogeneity, in the historical context of smaller underpowered studies, is a key factor complicating the search for replicable and generalizable BD biomarkers. For example, BD is highly heritable (~70%), with studies implicating a mosaic of common and rare genetic risk factors (Edvardsen et al., 2008; Toma et al., 2018). In the largest genome-wide association study to date (Stahl et al., 2019), thirty common genetic variants exceeding genome-wide significance thresholds were shown to increase the risk for BD. However, all of the genetic risk markers

combined account for only ~8% of the overall risk for BD. This indicates the presence of a high degree of polygenicity, hidden genetic signal and gene-environment interactions that have yet to be discovered.

The current funding landscape for bipolar disorder research has further exacerbated the gaps in the field. Using the search term “bipolar disorder” in the United States Federal Reporter database, the federal government has provided $2.1 billion in funding for bipolar disorder projects from fiscal year 2008 to 2018. The search term “schizophrenia” yielded more than $7 billion in funding, and $2.7 billion allocated to the term “major depressive disorder” in the same time frame. Searching the database for “bipolar disorder”, and excluding “schizophrenia” and “depression”, generates only $1.1 billion, which likely represents funding specific to bipolar disorder from 2008 to 2018. Notwithstanding the importance of research on MDD and schizophrenia (SCZ), it is vital to move the BD field forward by elucidating unique symptomatology, etiology, progression and targeted treatments. Furthermore, a recent report from the UK Mental Health Research Funding mental health charity MQ (Woelbert, 2019) found that less than 3% of public contributions from 2014-2017 in the UK went to mental health research. Even with support from non-government organizations to supplement federal funding, additional investment in BD research is required to investigate this complex disorder and achieve reliable and robust breakthroughs which will permit improved diagnosis and support better treatment and prognosis.

Classification of Bipolar Disorder (DSM-5)

Bipolar I Disorder:
Requires at least one manic episode. Typically includes depressive episodes but not required for diagnosis.

Bipolar II Disorder:
At least one hypomanic and one major depressive episode required for diagnosis.

Cyclothymic Disorder:
Depressive and hypomanic periods that do not meet criteria for major depression or hypomania for at least 2 years.

Other Specified bipolar and related disorder:
These disorders do not meet the criteria for BD-I, BD-II, or cyclothymia due to insufficient duration/severity of bipolar-like symptoms. For example, cyclothymia not lasting for 2 years, depressive episodes with short-duration hypomania, etc.

Unspecified bipolar and related disorder:
Other bipolar-like disorder including symptoms that do not meet full criteria for previous categories.

*Substance-, drug- or other medical condition-induced bipolar and related disorde*r:
Includes symptoms of bipolar disorder such as mania and mood instability that are caused by any medication, substance or medical condition. Examples include amphetamine or cocaine-induced mania and hyperthyroidism.

Specifiers:
Additional details that characterize symptoms including “with rapid cycling”, “with mixed features”, “with anxious distress”, etc.

*Note that the ENIGMA Bipolar Disorder Working Groups has performed analyses on BD-I, BD-II and other bipolar subtypes, including bipolar disorder not otherwise specified (BD-NOS), a subtype that was characterized by the 4th edition of the DSM, which has since been updated to “Other Specified” and “Unspecified” in the 5th edition of the DSM.

Ongoing ENIGMA Bipolar Disorder Working Group Projects

In-depth studies of altered brain morphometry, white matter connectivity and neurometabolites

Subcortical shape analysis

The ENIGMA Methods Core developed an advanced subcortical shape analysis technique in an effort to more finely map the spatial distribution of ROI-based alterations to subcortical structures reported in our initial study (Hibar et al., 2016). The ENIGMA Shape Analysis Pipeline (Gutman et al., 2015; http://enigma.ini.usc.edu/ongoing/enigma-shape-analysis/) derives local thickness

(radial distance) and surface area expansion/contraction (Jacobian determinant) measures across thousands of points along a subcortical structure's surface. The deficits in hippocampal, amygdala and thalamic volumes detected in our ROI-based analysis can now be interrogated on a more detailed level, revealing the topographic burden across underlying subfields or subcompartments with known structural and functional connectivity. Findings will provide more detailed mapping of case-control differences, as well as shape alterations associated with treatment, duration of illness and symptom severity. This technique may help guide more mechanistic investigations of distinct neuronal subpopulations in these structures and provide a better understanding of the complex subcortical pathophysiology in BD.

The ENIGMA Shape Analysis Pipeline has been applied more broadly across the ENIGMA clinical working groups and has revealed complex patterns of local variation in schizophrenia (Gutman et al., 2019), major depression (Ho et al., 2020 this edition), addiction (Chye et al., 2019) and 22q11.2 deletion syndrome (Ching et al., 2020). ENIGMA BD working group efforts are underway (see vertex-wise machine learning below) to directly compare subcortical shape metrics across disorders, as fine-grained mapping may serve to better differentiate overlapping gross volume effects seen across psychiatric disorders.

Mapping hippocampal subfield alterations

In ENIGMA's large-scale analyses of BD and SCZ, lower hippocampal volume is consistently one of the most prominent findings in affected individuals compared to healthy controls (Hibar et al., 2016; van Erp et al., 2016). Thus, the anatomically and functionally complex hippocampus could be a key target for understanding illness development and/or progression, but it is not clear how different hippocampal subfields are affected and how these finer-scale effects relate to BD pathophysiology.

Postmortem and *in vivo* MRI volumetric studies of distinct hippocampal subcomponents – e.g., the cornu ammonis, subiculum and dentate gyrus – show mixed results, with mostly smaller subfield volumes reported in BD (Harrison et al. 2018; Haukvik et al.,

2015; Haukvik et al., 2018). Indeed, a prior meta-analysis showed smaller volumes across all subfields in BD compared to healthy controls (Haukvik et al., 2018). To date, it has been difficult to reproduce findings across studies. Automated hippocampal subfield segmentation is inherently difficult and factors such as small sample sizes, clinical heterogeneity (Janiri et al., 2019), BD subtypes, illness stage, alcohol use, illicit substances, and medication can affect hippocampal structure.

Recently, through the development of an *ex-vivo* atlas, an automated hippocampal subfield segmentation algorithm for MRI scans was developed with improved accuracy (Iglesias et al., 2015), and made publicly available in FreeSurfer (version 6.0.0; http://www.freesurfer.net). ENIGMA has further developed standardized post-processing and quality control pipelines for hippocampus subfield segmentation that are used across different ENIGMA working groups. We recently submitted the largest hippocampal subfields analysis in BD to date, pooling data from over 4,600 MRI scans (1,472 individuals with BD and 3,226 HC) from 23 sites worldwide (Haukvik et al., submitted). The project is also modeling secondary effects of diagnostic subtype, medication use, and clinical characteristics on hippocampal subfield volumes and provides valuable insights into the impact of BD on hippocampus subcomponents in the largest study of its kind.

The role of obesity as a source of heterogeneity in bipolar disorder and underlying brain morphometry

While subtle brain imaging alterations are frequently associated with BD on a group level, such changes are found only in some, but not all BD individuals. This heterogeneity indicates that additional clinical factors likely play a role in BD pathogenesis. However, specific risk factors for neurostructural alterations in BD remain mostly unknown. One potential source of brain alterations in psychiatric disorders is the comorbidity with medical conditions known to affect the brain. Obesity, which is prevalent in BD, is associated with brain imaging alterations in frontal and mesiotemporal regions (Garcia-Garcia et al., 2019), similar to those observed in BD. A genetic link between increased BMI and BD is supported by a recent large-scale study

(Bahrami et al., 2020). We and others have demonstrated that obesity is an additional factor, which contributes to brain alterations in psychiatric disorders, including BD and first episode of SCZ (Bond et al., 2011, 2014; Kolenic et al., 2018). The presence or absence of obesity could explain why only some individuals with BD present with brain imaging alterations. Focusing on obesity may help to parse the neurostructural heterogeneity of BD and may hopefully help identify a modifiable risk factor for BD-related brain alterations.

Modeling polypharmacy effects on subcortical brain structures

A question that repeatedly emerges when considering the neuroanatomical deviations associated with BD is the extent to which psychotropic medications such as lithium and antipsychotics may be driving the neuroanatomical abnormalities identified in case-control studies. Substantial preclinical and clinical evidence points to lithium having neurotrophic effects (Quiroz et al, 2010, Hibar et al, 2018), whereas antipsychotic medications have been associated with reduced gray matter measures (Vernon et al, 2012; Ho et al, 2011; Hibar et al, 2018). In a previous mega-analysis of ROI segmentation studies of BD (Hallahan et al, 2011), there was a strong association between lithium use and enlargement of the hippocampus and amygdala. In the subsequent ENIGMA meta-analysis employing automated segmentation of subcortical volumes, lithium use was associated with enlarged thalamic volumes in individuals with BD. However, these and several other prior studies (Bearden et al., 2008) usually compared participants taking lithium to those who were not or to healthy volunteers, with only some assessing the impact of related clinical factors such as concurrent medications and treatment response (Hajek et al., 2012, 2014; Van Gestel et al., 2019). The purpose of this further research initiative is to conduct a more fine-grained analysis of medication effects and their potential modulators on subcortical brain volumes in a large cohort of BD participants at the individual level. Using the ENIGMA-standardized cortical and subcortical measures, we are applying linear mixed-effect models to map complex medication effects on brain structure while accounting for individual-level confounds. While the current study does not take the place of a randomized clinical trial,

the influence of polypharmacy (individuals taking multiple medications) and medication dose/duration on brain measures can provide important insights into BD treatment.

Network-based white matter connectivity

Building on decades of neuroanatomical, molecular and pharmacological studies on the underlying neurotransmitter systems at play in mood lability (Cade, 1949; Janowsky et al., 1972; Hajek et al, 2005; Strakowski et al., 2012), network-based connectivity (Bullmore & Sporns, 2009) has emerged as a powerful tool for studying complex behavioral, cognitive and clinical aspects of BD. White matter topology can be examined as maps of non-tensor based tractography weightings (Jeurissen et al., 2011) connecting cortical and subcortical brain regions. Previous studies have revealed altered brain integration and segregation in BD including connectivity deficits of fronto-limbic and basal ganglia regions (Perry et al., 2018; Nabulsi et al., 2019). However, such connectivity studies require high statistical power, especially when pursuing precise brain node definitions (Zalesky et al., 2010). As white matter connectivity graphs become more refined, thanks to increased data resolution and advanced diffusion acquisitions, data-driven and permutation-based corrections are critical to balance improved anatomical sensitivity with aforementioned limitations of previous, small-scale neuroimaging studies in BD (Zalesky et al., 2016). In an effort to better understand the underlying network-based mechanisms of mood instability in BD, we are currently applying these advanced connectivity analysis methods to the ENIGMA Bipolar Disorder Working Group diffusion-weighted samples.

Proton magnetic resonance spectroscopy (1H-MRS) for the analysis of brain metabolites

Proton magnetic resonance spectroscopy (1H-MRS) is a non-invasive technique used to measure brain metabolites *in vivo*, such as N-acetylaspartate, creatine (NAA), choline (Cho), glutamate (Glu), glutamine, glutamate+glutamine (Glx), myo-inositol (MI) and gamma aminobutyric acid (GABA) (Buonocore & Maddock, 2015). Despite the growing literature on 1H-MRS data in BD, previous systematic reviews and meta-analyses have mainly focused on specific neurometabolites such as Glu (Chitty et al., 2013; Gigante et

al., 2012; Yüksel & Ongur, 2010), GABA (Chiapponi et al., 2016; Romeo et al., 2018; Schür et al., 2016), Cho (Kraguljac et al., 2012; Yildiz-Yesiloglu & Ankerst, 2006) and MI (Silverstone et al., 2005). Given the historically small samples, results may have not been reported by brain region, mood state or account for complex factors such as medication.

To address these limitations, the ENIGMA Bipolar Disorder Spectroscopy Project is gathering data from research groups around the world to meta-analyze neurometabolite levels by mood episode, brain ROI (prefrontal, anterior cingulate, hippocampus, etc.) and examine associations with common comorbidities and medications. This effort represents one of the first multisite studies of brain metabolites across the ENIGMA consortium and is helping to inform future large-scale efforts of this kind.

Brain change and accelerated aging

Mapping longitudinal brain change

Although cross-sectional brain imaging studies show that BD is associated with structural brain abnormalities (Hibar et al., 2016, 2018; Hanford et al., 2016; Abe et al., 2016), it remains unknown whether such abnormalities represent static traits of BD or pathological changes over time. This is important, as the clinical course of BD appears to be progressive for some patients (Passos et al., 2016; Cardoso et al., 2015; Barbosa et al., 2014; Schneider et al., 2012). However, it has not been unambiguously established that brain structure actually changes during the course of illness in BD, beyond the changes that would be expected with normal aging (Najt et al. 2016; Weathers et al., 2018). This is because most brain imaging studies have been cross-sectional in design, whereas longitudinal studies are scarce and usually investigate smaller samples. The ENIGMA BD Working Group Longitudinal Brain Change Project aims to overcome these specific limitations.

By investigating cortical thickness, surface area, and subcortical volumes across two time points in a large, multicenter cohort of individuals with BD and HC, we aim to better understand the neuroprogressive nature of BD. We are employing a mega-analysis approach, using standardized ENIGMA protocols for longitudinal image processing with FreeSurfer (Reuter et al., 2012) in combination with linear mixed effects models (Bernel-Rusiel et al., 2013a, 2013b) and change rate analyses.

We expect that subgroups of individuals with BD will show abnormal changes in fronto-temporal cortices, as well as subcortical areas, such as the hippocampus and thalamus. Structural decline may be accelerated by the occurrence of manic episodes (Ekman et al., 2010) and may be countered by lithium use (Abe et al., 2015, Abe et al., 2019). By relating brain change to clinical variables, such as mood episodes and medication use, we hope to improve our understanding of the development and clinical trajectory of BD. Beyond this, future large-scale studies with multiple time-points per individual are needed to allow for modelling of nonlinear, individual-level trajectories of brain change.

Modeling accelerated brain aging

Many of the brain alterations discovered by the ENIGMA BD Working Group, when comparing BD with age-matched healthy individuals, are similar to those seen in typical aging (e.g., lower cortical thickens and hippocampal volume, decreased integrity of white matter microstructure, etc.). These findings, along with those from other age-related measures, have suggested that BD might involve advanced and/or accelerated aging processes (Rizzo et al., 2014). Using brain MRI measures, studies have begun to use machine learning approaches to estimate an individual’s ‘brain age’ and compare that to their chronological age, yielding a brain-predicted age difference (brain-PAD). Indications of apparent accelerated brain aging based on a larger brain-PAD have been found in several psychiatric disorders including SCZ, which may be related to cognitive decline (Franke & Gaser, 2019). Using ENIGMA-standardized brain measures, several ongoing studies have incorporated large samples of healthy control data to derive generalizable models of normative ‘brain age’ (Cole et al., 2017, 2019), using measures such as cortical thickness and surface area — brain metrics known to be under the

influence of differential neurodevelopmental genetic factors (Winkler et al., 2009; Panizzon et al., 2009).

In two forthcoming studies, the ENIGMA BD working group has collaborated with the ENIGMA MDD Working Group to develop a robust, multivariate estimator of brain age (Han et al., 2020). Measures of subcortical volume, cortical thickness and surface area were analyzed from 952 male and 1,236 female MDD controls using ridge regression to develop a model to predict chronological age. This model was validated within the MDD sample and then tested for generalizability to the ENIGMA BD Working Group's healthy controls to determine whether the mean absolute error (i.e., difference between chronological age and predicted brain age) was comparable to that of the HC participants from the MDD test sample. Final measures of Brain-PAD were calculated for the test samples. Importantly, the Brain-PAD models (separate for male and female) will be made available to the wider research community whereby users can derive Brain-PAD measures for their own research samples (https://old.photon-ai.com/enigma_brainage/).

Work is underway to evaluate the association between Brain-PAD measures and clinical characteristics within the ENIGMA BD Working Group including duration of illness, symptom severity and medication status. Working groups across the ENIGMA consortium are beginning to derive Brain-PAD measures based on this model, resulting in a number of future large-scale psychiatric brain age studies. Future work will include evaluating the extent of accelerated brain aging across a range of disorders as well as associations with polygenic risk (Kaufmann et al., 2019).

Advanced machine learning for improved classification and individual-level prediction

Vertex-wise machine learning

Previous machine learning on ROI-based measures has highlighted the potential of brain morphology to perform diagnostic classification of BD (Nunes et al., 2018).

Though classification accuracies based solely on engineered ROI features are promising, they fall short of the 80% positive predictive threshold sometimes thought to be clinically relevant (Savitz et al., 2013). One possible way forward is to use enhanced brain morphological features such as vertex-wise cortical and subcortical shape metrics, which provide thousands of local structural features across the brain. Such measures may include vertex-wise thickness, surface area, curvature, and sulcal features from the cerebral cortex. More detailed modeling of subcortical structures includes the analysis of vertex-wise radial distance (thickness), and the Jacobian determinant of the surface mesh (a measure of the surface 'dilation' or 'contraction' relative to an average template), as detailed above in the shape analysis section (Gutman et al., 2015). Relative to the ROI features, which average morphometric features across larger brain regions, vertex-wise metrics may improve discriminative power by providing finer-grained information on brain structure. However, using such detailed features comes at a cost, and the "curse of dimensionality" presents a challenge in pattern classification of such detailed features. The higher the sampling of the morphometric features, the smaller the distance is between individual sample points in the high-dimensional input feature space. The development of more advanced machine learning approaches using feature selection, representation learning and the application of deep learning may increase the accuracy of BD classification problems. Moreover, data driven clustering or biotyping (Drysdale et al., 2017; Dinga et al., 2019) may provide meaningful diagnostic subtypes that better correlate with symptom profiles and improve individual-level predictions.

Prediction of pharmacological treatment outcomes

In BD, optimal pharmacological treatment can vary between individuals and has even been shown to induce alternate mood states (e.g., antidepressants may induce mania). The creation of tools to aid physicians in matching patients with proper treatment is of utmost interest. The ENIGMA BD Working Group is currently exploring how ENIGMA-standardized MRI-derived brain features may be potentially applicable for possible clinical use including treatment selection.

Common medications for the treatment of BD have documented morphometric effects on brain structure (see main text) and the extent to which these brain features may provide insight into positive treatment responses is unknown. Of course, an optimal study of the relationship between brain measures and the adequacy of different treatment regimens would require a large-scale, longitudinal, randomized clinical trial. Here, we aim to provide some clues from an analysis of the large-scale cross-sectional dataset pooled by the ENIGMA BD Working Group and are training machine-learning models to predict which medications individuals with BD were prescribed at the time of their scan based on cortical and subcortical morphometric features. While limitations of such an approach are well recognized (e.g., cross-sectional design, the assumption that the medication at time of scan was an optimal treatment, etc.), using brain structure to predict treatment may provide much needed insights into brain features that might serve as potential biomarkers for tracking treatment response and guiding future targeted treatment strategies.

Multimodal classification and unsupervised clustering in multimodal MRI data

Supervised machine learning based on multiple MRI modalities may improve classification performance (Arbabshirani et al., 2017). Building off of the first machine learning analysis from the BD Working Group (Nunes et al., 2018), we are currently applying a similar strategy to classify BD individuals from controls using diffusion-weighted brain measures of white matter integrity (DTI) with the goal of incorporating resting-state fMRI (rsfMRI) data across sites. By fusing T1-based structural MRI, diffusion MRI and functional MRI data, we are working to apply unsupervised techniques (as forecast above) to derive biologically based clusters of individuals (Drysdale et al., 2017; Dinga et al., 2019) that may help tease apart BD heterogeneity in these large, multi-site samples.

Comparing bipolar brain morphometry and gene expression across psychiatric disorders

Multimodal structural and functional neuroimaging mediators of general intelligence across psychiatric disorders

Impairment in general cognitive ability (*g* factor) is a key feature among affective and psychotic disorders (e.g., MDD, BD and SCZ), and influences the functional status of patients (Rock et al. 2014; Bora et al., 2015; Green et al., 2016). General cognitive ability is particularly relevant since it represents the integration of independent brain processes working in concert to solve problems (Deary et al., 2010). Further, altered connectivity between brain regions is thought to underlie most psychiatric disorders (Baker et al., 2014; Zhang et al., 2011). ENIGMA has provided advances in understanding brain abnormalities in psychiatric disorders (van Erp et al. 2016; Hibar et al., 2016; Schmaal et al., 2016), but a valid criticism of well-powered neuroimaging studies in general is that their findings could be considered correlational. Different neuroimaging modalities are analyzed separately, despite shared variance among neuroimaging phenotypes, leading to difficulties in interpreting multimodal associations. The release of large scale and deeply phenotyped neuroimaging samples such as the human connectome project and the UK Biobank now allows for dissection of advanced multimodal neuroimaging phenotypes and their relationship with general cognitive ability at a whole-brain scale. We aim to probe mechanistic multimodal relationships among functional and structural MRI phenotypes that are associated with general cognitive ability in thousands of individuals using our TFCE mediation software (Lett et al., 2017). Utilizing the immense statistical power of the ENIGMA worldwide initiatives for examining MDD, BD, and SCZ, we aim to identify specific neuroimaging biomarkers that mediate reduced general cognitive ability and are specific to these disorders.

Virtual histology: Mapping cortical alterations to gene expression in bipolar disorder and beyond

The relationship between in vivo MRI and ex vivo histology is a key challenge facing neuroimaging research (Paus, 2018). Understanding this gap essential if we are to

understand how MRI-derived metrics relate to the underlying neurobiology. ENIGMA studies have revealed robust group differences in cortical thickness across several psychiatric illnesses, namely autism spectrum disorder (ASD), attention-deficit hyperactivity disorder (ADHD), BD, obsessive compulsive disorder (OCD), MDD and SCZ (Thompson et al., 2019). These macrostructural abnormalities within the cerebral cortex have yet to be fully understood at the level of histology and microstructure.

Multiple working groups within the ENIGMA consortium are currently working together to apply the “virtual histology” approach, associating structural brain metrics such as cortical thickness group differences to cell-specific gene expression from the Allen Human Brain Atlas (Hawrylycz et al. 2012; Patel et al. 2018; Shin et al. 2017). To ensure representativeness of the inter-regional profiles of gene expression, only genes that have passed filtering for donor-to-median correlation and similarity with another independent atlas of gene expression (BrainSpan) are used for subsequent analysis (Patel et al. 2018; Shin et al. 2017). Cell-specific gene lists are derived from single-cell RNA sequencing work characterizing pyramidal cells, interneurons, astrocytes, microglia, oligodendrocytes, endothelial cells, pericytes, and ependymal cells.

Group differences in cortical thickness across the 34 regions of the FreeSurfer Desikan-Killiany atlas (Desikan et al. 2006) are currently being generated meta-analytically from linear models applied across the ENIGMA working group adjusting for age, sex and site-specific covariates. The study sample includes over 12,000 cases and 15,000 healthy controls across the ENIGMA BD, ASD, ADHD, OCD, MDD and SCZ disorder working groups. Findings will help to better characterize the gene expression profiles underlying MRI-derived variations in cortical structure across these major neuropsychiatric disorders on an unprecedented scale.

Supplement References

Abe, C., Ekman, C. J., Sellgren, C., Petrovic, P., Ingvar, M., & Landen, M. (2016). Cortical thickness, volume and surface area in patients with bipolar disorder types I and II. J Psychiatry Neurosci, 41(4), 240-250. doi: 10.1503/jpn.150093

Abe, C., Ekman, C. J., Sellgren, C., Petrovic, P., Ingvar, M., & Landen, M. (2015). Manic episodes are related to changes in frontal cortex: a longitudinal neuroimaging study of bipolar disorder 1. Brain, 138(Pt 11), 3440-3448. doi: 10.1093/brain/awv266

Abe, C., Liberg, B., Song, J., Bergen, S. E., Petrovic, P., Ekman, C. J., . . . Landen, M. (2019). Longitudinal Cortical Thickness Changes in Bipolar Disorder and the Relationship to Genetic Risk, Mania, and Lithium Use. Biol Psychiatry. doi: 10.1016/j.biopsych.2019.08.015

Altamura, A. C., Dell'Osso, B., Berlin, H. A., Buoli, M., Bassetti, R., & Mundo, E. (2010). Duration of untreated illness and suicide in bipolar disorder: a naturalistic study. Eur Arch Psychiatry Clin Neurosci, 260(5), 385-391. doi: 10.1007/s00406-009-0085-2

Arbabshirani, M. R., Plis, S., Sui, J., & Calhoun, V. D. (2017). Single subject prediction of brain disorders in neuroimaging: Promises and pitfalls. Neuroimage, 145(Pt B), 137-165. doi: 10.1016/j.neuroimage.2016.02.079

Bahrami, S., Steen, N. E., Shadrin, A., O'Connell, K., Frei, O., Bettella, F., . . . Andreassen, O. A. (2020). Shared Genetic Loci Between Body Mass Index and Major Psychiatric Disorders: A Genome-wide Association Study. Jama Psychiatry. doi:10.1001/jamapsychiatry.2019.4188

Baker, J. T., Holmes, A. J., Masters, G. A., Yeo, B. T., Krienen, F., Buckner, R. L., & Ongur, D. (2014). Disruption of cortical association networks in schizophrenia and psychotic bipolar disorder. JAMA Psychiatry, 71(2), 109-118. doi: 10.1001/jamapsychiatry.2013.3469

Barbosa, I. G., Bauer, M. E., Machado-Vieira, R., & Teixeira, A. L. (2014). Cytokines in bipolar disorder: paving the way for neuroprogression. Neural Plast, 2014, 360481. doi: 10.1155/2014/360481

Bearden, C. E., Thompson, P. M., Dutton, R. A., Frey, B. N., Peluso, M. A., Nicoletti, M., . . . Soares, J. C. (2008). Three-dimensional mapping of hippocampal anatomy in unmedicated and lithium-treated patients with bipolar disorder. Neuropsychopharmacology, 33(6), 1229-1238. doi: 10.1038/sj.npp.1301507

Berk, M., Dodd, S., Callaly, P., Berk, L., Fitzgerald, P., de Castella, A. R., . . . Kulkarni, J. (2007). History of illness prior to a diagnosis of bipolar disorder or schizoaffective disorder. J Affect Disord, 103(1-3), 181-186. doi: 10.1016/j.jad.2007.01.027

Bernal-Rusiel, J. L., Reuter, M., Greve, D. N., Fischl, B., Sabuncu, M. R., & Alzheimer's Disease Neuroimaging, I. (2013). Spatiotemporal linear mixed effects modeling for the mass-univariate analysis of longitudinal neuroimage data. Neuroimage, 81, 358-370. doi: 10.1016/j.neuroimage.2013.05.049

Bernal-Rusiel, J. L., Greve, D. N., Reuter, M., Fischl, B., Sabuncu, M. R., & Alzheimer's Disease Neuroimaging, I. (2013). Statistical analysis of longitudinal neuroimage data with Linear Mixed Effects models. Neuroimage, 66, 249-260. doi: 10.1016/j.neuroimage.2012.10.065

Bond, D. J., Ha, T. H., Lang, D. J., Su, W., Torres, I. J., Honer, W. G., . . . Yatham, L. N. (2014). Body mass index-related regional gray and white matter volume reductions in first-episode mania patients. Biol Psychiatry, 76(2), 138-145. doi: 10.1016/j.biopsych.2013.08.030

Bond, D. J., Lang, D. J., Noronha, M. M., Kunz, M., Torres, I. J., Su, W., . . . Yatham, L. N. (2011). The association of elevated body mass index with reduced brain volumes in first-episode mania. Biol Psychiatry, 70(4), 381-387. doi: 10.1016/j.biopsych.2011.02.025

Bourne, C., Aydemir, O., Balanza-Martinez, V., Bora, E., Brissos, S., Cavanagh, J. T., . . . Goodwin, G. M. (2013). Neuropsychological testing of cognitive impairment in euthymic bipolar disorder: an individual patient data meta-analysis. Acta Psychiatr Scand, 128(3), 149-162. doi: 10.1111/acps.12133

Bora, E. (2015). Developmental trajectory of cognitive impairment in bipolar disorder: comparison with schizophrenia. Eur Neuropsychopharmacol, 25(2), 158-168. doi:10.1016/j.euroneuro.2014.09.007

Buonocore, M. H., & Maddock, R. J. (2015). Magnetic resonance spectroscopy of the brain: a review of physical principles and technical methods. Reviews in the Neurosciences, 26(6), 609–632. http://doi.org/10.1515/revneuro-2015-0010

Bullmore, E., & Sporns, O. (2009). Complex brain networks: graph theoretical analysis of structural and functional systems. Nat Rev Neurosci, 10(3), 186–198. doi: https://doi.org/10.1038/nrn2575

Cade, J. F. (1949). Lithium Salts in the Treatment of Psychotic Excitement. Med. J. Austral., (2), 349–352. https://doi.org/10.5694/j.1326-5377.1949.tb36912.x

Cardoso, T., Bauer, I. E., Meyer, T. D., Kapczinski, F., & Soares, J. C. (2015). Neuroprogression and Cognitive Functioning in Bipolar Disorder: A Systematic Review. Curr Psychiatry Rep, 17(9), 75. doi: 10.1007/s11920-015-0605-x

Chiapponi, C., Piras, F., Piras, F., Caltagirone, C., & Spalletta, G. (2016). GABA System in Schizophrenia and Mood Disorders: A Mini Review on Third-Generation Imaging Studies. Frontiers in Psychiatry, 7, 61. http://doi.org/10.3389/fpsyt.2016.00061

Ching, C. R. K., Gutman, B. A., Sun, D., Villalon Reina, J., Ragothaman, A., Isaev, D., . . . Bearden, C. E. (2020). Mapping Subcortical Brain Alterations in 22q11.2 Deletion Syndrome: Effects of Deletion Size and Convergence With Idiopathic Neuropsychiatric Illness. Am J Psychiatry, 177(7), 589-600. doi:10.1176/appi.ajp.2019.19060583

Chitty, K. M., Lagopoulos, J., Lee, R. S. C., Hickie, I. B., & Hermens, D. F. (2013). A systematic review and meta-analysis of proton magnetic resonance spectroscopy and mismatch negativity in bipolar disorder. European Neuropsychopharmacology : the Journal of the European College of Neuropsychopharmacology, 23(11), 1348–1363. http://doi.org/10.1016/j.euroneuro.2013.07.007

Chye, Y., Mackey, S., Gutman, B. A., Ching, C. R. K., Batalla, A., Blaine, S., . . . Garavan, H. (2019). Subcortical surface morphometry in substance dependence: An ENIGMA addiction working group study. Addict Biol, e12830. doi: 10.1111/adb.12830

Cole, J. H., & Franke, K. (2017). Predicting Age Using Neuroimaging: Innovative Brain Ageing Biomarkers. Trends Neurosci, 40(12), 681-690. doi: 10.1016/j.tins.2017.10.001

Cole, J. H., Marioni, R. E., Harris, S. E., & Deary, I. J. (2019). Brain age and other bodily 'ages': implications for neuropsychiatry. Mol Psychiatry, 24(2), 266-281. doi: 10.1038/s41380-018-0098-1

Deary, I. J., Penke, L., & Johnson, W. (2010). The neuroscience of human intelligence differences. Nat Rev Neurosci, 11(3), 201-211. doi: 10.1038/nrn2793

Depp, C. A., Mausbach, B. T., Harmell, A. L., Savla, G. N., Bowie, C. R., Harvey, P. D., & Patterson, T. L. (2012). Meta-analysis of the association between cognitive abilities and everyday functioning in bipolar disorder. Bipolar Disord, 14(3), 217-226. doi: 10.1111/j.1399-5618.2012.01011.x

Desikan, R. S., Ségonne, F., Fischl, B., Quinn, B. T., Dickerson, B. C., Blacker, D., ... & Albert, M. S. (2006). An automated labeling system for subdividing the human cerebral cortex on MRI scans into gyral based regions of interest. Neuroimage, 31(3), 968-980.

Dinga, R., Schmaal, L., Penninx, B., van Tol, M. J., Veltman, D. J., van Velzen, L., . . . Marquand, A. F. (2019). Evaluating the evidence for biotypes of depression: Methodological replication and extension of. Neuroimage Clin, 22, 101796. doi:10.1016/j.nicl.2019.101796

Drysdale, A. T., Grosenick, L., Downar, J., Dunlop, K., Mansouri, F., Meng, Y., . . . Liston, C. (2017). Resting-state connectivity biomarkers define neurophysiological subtypes of depression. Nat Med, 23(1), 28-38. doi: 10.1038/nm.4246

Edvardsen, J., Torgersen, S., Roysamb, E., Lygren, S., Skre, I., Onstad, S., & Oien, P. A. (2008). Heritability of bipolar spectrum disorders. Unity or heterogeneity? J Affect Disord, 106(3), 229-240. doi: 10.1016/j.jad.2007.07.001

Ekman, C. J., Lind, J., Ryden, E., Ingvar, M., & Landen, M. (2010). Manic episodes are associated with grey matter volume reduction - a voxel-based morphometry brain analysis. Acta Psychiatr Scand, 122(6), 507-515. doi: 10.1111/j.1600-0447.2010.01586.x

Franke, K., & Gaser, C. (2019). Ten Years of BrainAGE as a Neuroimaging Biomarker of Brain Aging: What Insights Have We Gained? Front Neurol, 10, 789. doi: 10.3389/fneur.2019.00789

Garcia-Garcia, I., Michaud, A., Dadar, M., Zeighami, Y., Neseliler, S., Collins, D. L., . . . Dagher, A. (2019). Neuroanatomical differences in obesity: meta-analytic findings and their validation in an independent dataset. Int J Obes (Lond), 43(5), 943-951. doi: 10.1038/s41366-018-0164-4

Gigante, A. D., Bond, D. J., Lafer, B., Lam, R. W., Young, L. T., & Yatham, L. N. (2012). Brain glutamate levels measured by magnetic resonance spectroscopy in patients with bipolar disorder: a meta-analysis. Bipolar Disorders, 14(5), 478–487. http://doi.org/10.1111/j.1399-5618.2012.01033.x

Gitlin, M. J., Swendsen, J., Heller, T. L., & Hammen, C. (1995). Relapse and impairment in bipolar disorder. Am J Psychiatry, 152(11), 1635-1640. doi: 10.1176/ajp.152.11.1635

Goldberg, J. F., Harrow, M., & Whiteside, J. E. (2001). Risk for bipolar illness in patients initially hospitalized for unipolar depression. Am J Psychiatry, 158(8), 1265-1270. doi: 10.1176/appi.ajp.158.8.1265

Goodwin, G. M., Anderson, I., Arango, C., Bowden, C. L., Henry, C., Mitchell, P. B., . . . Wittchen, H. U. (2008). ECNP consensus meeting. Bipolar depression. Nice, March 2007. Eur Neuropsychopharmacol, 18(7), 535-549. doi: 10.1016/j.euroneuro.2008.03.003

Grande, I., Berk, M., Birmaher, B., & Vieta, E. (2016). Bipolar disorder. Lancet, 387(10027), 1561-1572. doi: 10.1016/S0140-6736(15)00241-X\

Green, M. F. (2016). Impact of cognitive and social cognitive impairment on functional outcomes in patients with schizophrenia. J Clin Psychiatry, 77 Suppl 2, 8-11. doi: 10.4088/JCP.14074su1c.02

Gutman, B. A., Fletcher, P. T., Cardoso, M. J., Fleishman, G. M., Lorenzi, M., Thompson, P. M., & Ourselin, S. (2015). A Riemannian Framework for Intrinsic Comparison of Closed Genus-Zero Shapes. Inf Process Med Imaging, 24, 205-218. doi: 10.1007/978-3-319-19992-4_16

Gutman, B. A., van Erp T. G. M., Alber, K., Ching, C. R. K., Isaev, D., Jahanshad, N., . . . Wang, L. (2019). Meta-analysis of deep brain structural shape and asymmetry in schizophrenia via ENIGMA. Submitted to Human Brain Mapping.

Hajek, T., Bauer, M., Pfennig, A., Cullis, J., Ploch, J., O'Donovan, C., . . . Alda, M. (2012). Large positive effect of lithium on prefrontal cortex N-acetylaspartate in patients with bipolar disorder: 2-centre study. J Psychiatry Neurosci, 37(3), 185-192. doi: 10.1503/jpn.110097

Hajek, T., Calkin, C., Blagdon, R., Slaney, C., Uher, R., & Alda, M. (2014). Insulin resistance, diabetes mellitus, and brain structure in bipolar disorders. Neuropsychopharmacology, 39(12), 2910-2918. doi: 10.1038/npp.2014.148

Hajek, T., Carrey, N. and Alda, M. (2005), Neuroanatomical abnormalities as risk factors for bipolar disorder. Bipolar Disorders, 7: 393-403. doi: https://doi.org/10.1111/j.1399-5618.2005.00238.x

Hajek, T., Kopecek, M., Hoschl, C., & Alda, M. (2012). Smaller hippocampal volumes in patients with bipolar disorder are masked by exposure to lithium: a meta-analysis. J Psychiatry Neurosci, 37(5), 333-343. doi: 10.1503/jpn.110143

Hallahan, B., Newell, J., Soares, J. C., Brambilla, P., Strakowski, S. M., Fleck, D. E., . . . McDonald, C. (2011). Structural magnetic resonance imaging in bipolar disorder: an international collaborative mega-analysis of individual adult patient data. Biol Psychiatry, 69(4), 326-335. doi: 10.1016/j.biopsych.2010.08.029

Han, L. K. M., Dinga, R., Hahn, T., Ching, C. R. K., Eyler, L. T., Aftanas, L., . . . Schmaal, L. (2020). Brain aging in major depressive disorder: results from the ENIGMA major depressive disorder working group. Mol Psychiatry. doi:10.1038/s41380-020-0754-0

Hanford, L. C., Nazarov, A., Hall, G. B., & Sassi, R. B. (2016). Cortical thickness in bipolar disorder: a systematic review. Bipolar Disord, 18(1), 4-18. doi: 10.1111/bdi.12362

Harrison, P. J., Colbourne, L., & Harrison, C. H. (2018). The neuropathology of bipolar disorder: Systematic review and meta-analysis. Molecular Psychiatry, 1–22. https://doi.org/10.1038/s41380-018-0213-3

Haukvik, U. K., Westlye, L. T., Morch-Johnsen, L., Jorgensen, K. N., Lange, E. H., Dale, A. M., … Agartz, I. (2015). In vivo hippocampal subfield volumes in schizophrenia and bipolar disorder. Biological Psychiatry, 77(6), 581–588. https://doi.org/10.1016/j.biopsych.2014.06.020

Haukvik, Unn K., Tamnes, C. K., Söderman, E., & Agartz, I. (2018). Neuroimaging hippocampal subfields in schizophrenia and bipolar disorder: A systematic review and meta-analysis. Journal of Psychiatric Research, 104, 217–226. https://doi.org/10.1016/j.jpsychires.2018.08.012

Haukvik, U. K., Gurholt, T. P., Nerland, S., Elvsåshagen, T., Akudjedu, T. N., Alda, M., … for the ENIGMA Bipolar Disorder Working group. (2020). In vivo hippocampal subfield volumes in bipolar disorder – A mega-analysis from the ENIGMA consortium. Submitted to Human Brain Mapping

Hawrylycz, M. J., Lein, E. S., Guillozet-Bongaarts, A. L., Shen, E. H., Ng, L., Miller, J. A., ... & Abajian, C. (2012). An anatomically comprehensive atlas of the adult human brain transcriptome. Nature, 489(7416), 391.

Hibar, D. P., Westlye, L. T., van Erp, T. G., Rasmussen, J., Leonardo, C. D., Faskowitz, J., . . . Andreassen, O. A. (2016). Subcortical volumetric abnormalities in bipolar disorder. Mol Psychiatry, 21(12), 1710-1716. doi: 10.1038/mp.2015.227

Hibar, D. P., Westlye, L. T., Doan, N. T., Jahanshad, N., Cheung, J. W., Ching, C. R. K., . . . Andreassen, O. A. (2018). Cortical abnormalities in bipolar disorder: an MRI analysis of 6503 individuals from the ENIGMA Bipolar Disorder Working Group. Mol Psychiatry, 23(4), 932-942. doi: 10.1038/mp.2017.73

Ho, B. C., Andreasen, N. C., Ziebell, S., Pierson, R., & Magnotta, V. (2011). Long-term antipsychotic treatment and brain volumes: a longitudinal study of first-episode schizophrenia. Arch Gen Psychiatry, 68(2), 128-137. doi: 10.1001/archgenpsychiatry.2010.199

Ho, T. C., Gutman, B., Pozzi, E., Grabe, H. J., Hosten, N., Wittfeld, K., . . . Schmaal, L. (2020). Subcortical shape alterations in major depressive disorder: Findings from the ENIGMA major depressive disorder working group. Human Brain Mapping. doi:10.1002/hbm.24988

Iglesias, J. E., Augustinack, J. C., Nguyen, K., Player, C. M., Player, A., Wright, M., … Alzheimer's Disease Neuroimaging, I. (2015). A computational atlas of the hippocampal formation using ex vivo, ultra-high resolution MRI: Application to adaptive segmentation of in vivo MRI. NeuroImage, 115, 117–137. https://doi.org/10.1016/j.neuroimage.2015.04.042

Janiri, D., Simonetti, A., Piras, F., Ciullo, V., Spalletta, G., & Sani, G. (2019). Predominant polarity and hippocampal subfield volumes in Bipolar disorders. Bipolar Disorders, n/a(n/a). https://doi.org/10.1111/bdi.12857

Janowsky, D. S., Davis, J. M., El-Yousef, M. K., & Sekerke, H. J. (1972). a Cholinergic-Adrenergic Hypothesis of Mania and Depression. The Lancet, 300(7778), 632–635. doi: https://doi.org/10.1016/S0140-6736(72)93021-8

Jeurissen, B., Leemans, A., Jones, D. K., Tournier, J., & Sijbers, J. (2011). Probabilistic Fiber Tracking Using the Residual Bootstrap with Constrained Spherical Deconvolution, 479, 461–479. doi: https://doi.org/10.1002/hbm.21032

Judd, L. L., & Akiskal, H. S. (2003). The prevalence and disability of bipolar spectrum disorders in the US population: re-analysis of the ECA database taking into account subthreshold cases. J Affect Disord, 73(1-2), 123-131. doi: 10.1016/s0165-0327(02)00332-4

Kaufmann, T., van der Meer, D., Doan, N. T., Schwarz, E., Lund, M. J., Agartz, I., . . . Westlye, L. T. (2019). Common brain disorders are associated with heritable patterns of apparent aging of the brain. Nat Neurosci, 22(10), 1617-1623. doi: 10.1038/s41593-019-0471-7

Keck, P. E., Jr., Kessler, R. C., & Ross, R. (2008). Clinical and economic effects of unrecognized or inadequately treated bipolar disorder. J Psychiatr Pract, 14 Suppl 2, 31-38. doi: 10.1097/01.pra.0000320124.91799.2a

Kolenic, M., Franke, K., Hlinka, J., Matejka, M., Capkova, J., Pausova, Z., . . . Hajek, T. (2018). Obesity, dyslipidemia and brain age in first-episode psychosis. J Psychiatr Res, 99, 151-158. doi: 10.1016/j.jpsychires.2018.02.012

Kozicky, J. M., Torres, I. J., Silveira, L. E., Bond, D. J., Lam, R. W., & Yatham, L. N. (2014). Cognitive change in the year after a first manic episode: association between clinical outcome and cognitive performance early in the course of bipolar I disorder. J Clin Psychiatry, 75(6), e587-593. doi: 10.4088/JCP.13m08928

Kraguljac, N. V., Reid, M., White, D., Jones, R., Hollander, den, J., Lowman, D., & Lahti, A. C. (2012). Neurometabolites in schizophrenia and bipolar disorder - a systematic review and meta-analysis. Psychiatry Research, 203(2-3), 111–125. http://doi.org/10.1016/j.pscychresns.2012.02.003

Lewandowski, K. E., Sperry, S. H., Cohen, B. M., Norris, L. A., Fitzmaurice, G. M., Ongur, D., & Keshavan, M. S. (2017). Treatment to Enhance Cognition in Bipolar Disorder (TREC-BD): Efficacy of a Randomized Controlled Trial of Cognitive Remediation Versus Active Control. J Clin Psychiatry, 78(9), e1242-e1249. doi: 10.4088/JCP.17m11476

Lett, T. A., Waller, L., Tost, H., Veer, I. M., Nazeri, A., Erk, S., . . . Walter, H. (2017). Cortical surface-based threshold-free cluster enhancement and cortexwise mediation. Hum Brain Mapp, 38(6), 2795-2807. doi: 10.1002/hbm.23563

Martinez-Aran, A., & Vieta, E. (2015). Cognition as a target in schizophrenia, bipolar disorder and depression. Eur Neuropsychopharmacol, 25(2), 151-157. doi: 10.1016/j.euroneuro.2015.01.007

Nabulsi, L., McPhilemy, G., Kilmartin, L., O'Hora, D., O'Donoghue, S., Forcellini, G., . . . Cannon, D. M. (2019). Bipolar Disorder and Gender are Associated with Fronto-limbic and Basal Ganglia Dysconnectivity: A Study of Topological Variance Using Network Analysis. Brain Connectivity. doi: https://doi.org/10.1089/brain.2019.0667

Najt, P., Wang, F., Spencer, L., Johnston, J. A., Cox Lippard, E. T., Pittman, B. P., . . . Blumberg, H. P. (2016). Anterior Cortical Development During Adolescence in Bipolar Disorder. Biol Psychiatry, 79(4), 303-310. doi: 10.1016/j.biopsych.2015.03.026

Nunes, A., Schnack, H. G., Ching, C. R. K., Agartz, I., Akudjedu, T. N., Alda, M., . . . Group, E. B. D. W. (2018). Using structural MRI to identify bipolar disorders - 13 site machine learning study in 3020 individuals from the ENIGMA Bipolar Disorders Working Group. Mol Psychiatry. doi: 10.1038/s41380-018-0228-9

Panizzon, M. S., Fennema-Notestine, C., Eyler, L. T., Jernigan, T. L., Prom-Wormley, E., Neale, M., . . . Kremen, W. S. (2009). Distinct genetic influences on cortical surface area and cortical thickness. Cereb Cortex, 19(11), 2728-2735. doi: 10.1093/cercor/bhp026

Patel, Y., Shin, J., Gowland, P. A., Pausova, Z., Paus, T., & IMAGEN consortium. (2018). Maturation of the human cerebral cortex during adolescence: myelin or dendritic arbor? Cerebral Cortex, 29(8), 3351-3362.

Passos, I. C., Ballester, P. L., Barros, R. C., Librenza-Garcia, D., Mwangi, B., Birmaher, B., . . . Kapczinski, F. (2019). Machine learning and big data analytics in bipolar disorder: A position paper from the International Society for Bipolar Disorders Big Data Task Force. Bipolar Disord, 21(7), 582-594. doi: 10.1111/bdi.12828

Passos, I. C., Mwangi, B., Vieta, E., Berk, M., & Kapczinski, F. (2016). Areas of controversy in neuroprogression in bipolar disorder. Acta Psychiatr Scand, 134(2), 91-103. doi: 10.1111/acps.12581

Paus, T. (2018). Imaging microstructure in the living human brain: A viewpoint. Neuroimage, 182, 3-7. doi: 10.1016/j.neuroimage.2017.10.013

Perlis, R. H., Ostacher, M. J., Patel, J. K., Marangell, L. B., Zhang, H., Wisniewski, S. R., . . . Thase, M. E. (2006). Predictors of recurrence in bipolar disorder: primary outcomes from the Systematic Treatment Enhancement Program for Bipolar Disorder (STEP-BD). Am J Psychiatry, 163(2), 217-224. doi: 10.1176/appi.ajp.163.2.217

Perry, A., Roberts, G., Mitchell, P. B., & Breakspear, M. (2018). Connectomics of bipolar disorder: a critical review, and evidence for dynamic instabilities within interoceptive networks. Molecular Psychiatry. doi: https://doi.org/10.1038/s41380-018-0267-2

Pompili, M., Gonda, X., Serafini, G., Innamorati, M., Sher, L., Amore, M., . . . Girardi, P. (2013). Epidemiology of suicide in bipolar disorders: a systematic review of the literature. Bipolar Disord, 15(5), 457-490. doi: 10.1111/bdi.12087

Quiroz, J. A., Machado-Vieira, R., Zarate, C. A., Jr., & Manji, H. K. (2010). Novel insights into lithium's mechanism of action: neurotrophic and neuroprotective effects. Neuropsychobiology, 62(1), 50-60. doi: 10.1159/000314310

Reuter, M., Schmansky, N. J., Rosas, H. D., & Fischl, B. (2012). Within-subject template estimation for unbiased longitudinal image analysis. Neuroimage, 61(4), 1402-1418. doi: 10.1016/j.neuroimage.2012.02.084

Rizzo, L. B., Costa, L. G., Mansur, R. B., Swardfager, W., Belangero, S. I., Grassi-Oliveira, R., . . . Brietzke, E. (2014). The theory of bipolar disorder as an illness of accelerated aging: implications for clinical care and research. Neurosci Biobehav Rev, 42, 157-169. doi: 10.1016/j.neubiorev.2014.02.004

Rock, P. L., Roiser, J. P., Riedel, W. J., & Blackwell, A. D. (2014). Cognitive impairment in depression: a systematic review and meta-analysis. Psychol Med, 44(10), 2029-2040. doi: 10.1017/S0033291713002535

Romeo, B., Choucha, W., Fossati, P., & Rotge, J.-Y. (2018). Meta-analysis of central and peripheral γ-aminobutyric acid levels in patients with unipolar and bipolar depression. Journal of Psychiatry & Neuroscience : JPN, 43(1), 58–66.

Rosa, A. R., Gonzalez-Ortega, I., Gonzalez-Pinto, A., Echeburua, E., Comes, M., Martinez-Aran, A., . . . Vieta, E. (2012). One-year psychosocial functioning in patients in the early vs. late stage of bipolar disorder. Acta Psychiatr Scand, 125(4), 335-341. doi: 10.1111/j.1600-0447.2011.01830.x

Sanchez-Moreno, J., Bonnin, C., Gonzalez-Pinto, A., Amann, B. L., Sole, B., Balanza-Martinez, V., . . . Group, C. F. R. (2017). Do patients with bipolar disorder and subsyndromal symptoms benefit from functional remediation? A 12-month follow-up study. Eur Neuropsychopharmacol, 27(4), 350-359. doi: 10.1016/j.euroneuro.2017.01.010

Savitz, J. B., Rauch, S. L., & Drevets, W. C. (2013). Clinical application of brain imaging for the diagnosis of mood disorders: the current state of play. Mol Psychiatry, 18(5), 528-539. doi: 10.1038/mp.2013.25

Schaffer, A., Isometsa, E. T., Tondo, L., D, H. M., Turecki, G., Reis, C., . . . Yatham, L. N. (2015). International Society for Bipolar Disorders Task Force on Suicide: meta-analyses and meta-regression of correlates of suicide attempts and suicide deaths in bipolar disorder. Bipolar Disord, 17(1), 1-16. doi: 10.1111/bdi.12271

Schmaal, L., Veltman, D. J., van Erp, T. G., Samann, P. G., Frodl, T., Jahanshad, N., . . . Hibar, D. P. (2016). Subcortical brain alterations in major depressive disorder: findings from the ENIGMA Major Depressive Disorder working group. Mol Psychiatry, 21(6), 806-812. doi: 10.1038/mp.2015.69

Schneider, M. R., DelBello, M. P., McNamara, R. K., Strakowski, S. M., & Adler, C. M. (2012). Neuroprogression in bipolar disorder. Bipolar Disord, 14(4), 356-374. doi: 10.1111/j.1399-5618.2012.01024.x

Schür, R. R., Draisma, L. W. R., Wijnen, J. P., Boks, M. P., Koevoets, M. G. J. C., Joëls, M., et al. (2016). Brain GABA levels across psychiatric disorders: A systematic literature review and meta-analysis of (1) H-MRS studies. Human Brain Mapping, 37(9), 3337–3352. http://doi.org/10.1002/hbm.23244

Shin, J., French, L., Xu, T., Leonard, G., Perron, M., Pike, G. B., ... & Paus, T. (2017). Cell-specific gene-expression profiles and cortical thickness in the human brain. Cerebral Cortex, 28(9), 3267-3277.

Silverstone, P. H., McGrath, B. M., & Kim, H. (2005). Bipolar disorder and myo-inositol: a review of the magnetic resonance spectroscopy findings. Bipolar Disorders, 7(1), 1–10. http://doi.org/10.1111/j.1399-5618.2004.00174.x

Solomon, D. A., Keitner, G. I., Miller, I. W., Shea, M. T., & Keller, M. B. (1995). Course of illness and maintenance treatments for patients with bipolar disorder. J Clin Psychiatry, 56(1), 5-13.

Sparding, T., Silander, K., Palsson, E., Ostlind, J., Sellgren, C., Ekman, C. J., . . . Landen, M. (2015). Cognitive functioning in clinically stable patients with bipolar disorder I and II. PLoS One, 10(1), e0115562. doi: 10.1371/journal.pone.0115562

Sparding, T., Silander, K., Palsson, E., Ostlind, J., Ekman, C. J., Sellgren, C. M., . . . Landen, M. (2017). Classification of cognitive performance in bipolar disorder. Cogn Neuropsychiatry, 22(5), 407-421. doi: 10.1080/13546805.2017.1361391

Stahl, E. A., Breen, G., Forstner, A. J., McQuillin, A., Ripke, S., Trubetskoy, V., . . . Bipolar Disorder Working Group of the Psychiatric Genomics, C. (2019). Genome-wide association study identifies 30 loci associated with bipolar disorder. Nat Genet, 51(5), 793-803. doi: 10.1038/s41588-019-0397-8

Strakowski, S. M., Adler, C. M., Almeida, J., Altshuler, L. L., Blumberg, H. P., Chang, K. D., … Townsend, J. D. (2012). The functional neuroanatomy of bipolar disorder: A consensus model. Bipolar Disorders, 14(4), 313–325. doi: https://doi.org/10.1111/j.1399-5618.2012.01022.x

Thompson, P. M., Jahanshad, N., Ching, C. R. K., Salminen, L. E., Thomopoulos, S., . . . PsyArXiv Preprints (2019). ENIGMA and Global Neuroscience: A Decade of Large-Scale Studies of the Brain in Health and Disease across more than 40 Countries as a neural correlate of treatment. doi: 10.31234/osf.io/qnsh7

Toma, C., Shaw, A. D., Allcock, R. J. N., Heath, A., Pierce, K. D., Mitchell, P. B., . . . Fullerton, J. M. (2018). An examination of multiple classes of rare variants in extended families with bipolar disorder. Transl Psychiatry, 8(1), 65. doi: 10.1038/s41398-018-0113-y

Torres, I. J., DeFreitas, V. G., DeFreitas, C. M., Kauer-Sant'Anna, M., Bond, D. J., Honer, W. G., . . . Yatham, L. N. (2010). Neurocognitive functioning in patients with bipolar I disorder recently recovered from a first manic episode. J Clin Psychiatry, 71(9), 1234-1242. doi: 10.4088/JCP.08m04997yel

Vazquez, G. H., Holtzman, J. N., Lolich, M., Ketter, T. A., & Baldessarini, R. J. (2015). Recurrence rates in bipolar disorder: Systematic comparison of long-term prospective, naturalistic studies versus randomized controlled trials. Eur Neuropsychopharmacol, 25(10), 1501-1512. doi: 10.1016/j.euroneuro.2015.07.013

van Erp, T. G., Hibar, D. P., Rasmussen, J. M., Glahn, D. C., Pearlson, G. D., Andreassen, O. A., . . . Turner, J. A. (2016). Subcortical brain volume abnormalities in 2028 individuals with schizophrenia and 2540 healthy controls via the ENIGMA consortium. Mol Psychiatry, 21(4), 585. doi: 10.1038/mp.2015.118

Van Gestel, H., Franke, K., Petite, J., Slaney, C., Garnham, J., Helmick, C., . . . Hajek, T. (2019). Brain age in bipolar disorders: Effects of lithium treatment. Aust N Z J Psychiatry, 53(12), 1179-1188. doi: 10.1177/0004867419857814

Vieta, E., Berk, M., Schulze, T. G., Carvalho, A. F., Suppes, T., Calabrese, J. R., . . . Grande, I. (2018). Bipolar disorders. Nat Rev Dis Primers, 4, 18008. doi: 10.1038/nrdp.2018.8

Vernon, A. C., Natesan, S., Crum, W. R., Cooper, J. D., Modo, M., Williams, S. C., & Kapur, S. (2012). Contrasting effects of haloperidol and lithium on rodent brain structure: a magnetic resonance imaging study with postmortem confirmation. Biol Psychiatry, 71(10), 855-863. doi: 10.1016/j.biopsych.2011.12.004

Weathers, J., Lippard, E. T. C., Spencer, L., Pittman, B., Wang, F., & Blumberg, H. P. (2018). Longitudinal Diffusion Tensor Imaging Study of Adolescents and Young Adults With Bipolar Disorder. J Am Acad Child Adolesc Psychiatry, 57(2), 111-117. doi: 10.1016/j.jaac.2017.11.014

Winkler, A. M., Kochunov, P., Blangero, J., Almasy, L., Zilles, K., Fox, P. T., . . . Glahn, D. C. (2010). Cortical thickness or grey matter volume? The importance of selecting the phenotype for imaging genetics studies. Neuroimage, 53(3), 1135-1146. doi: 10.1016/j.neuroimage.2009.12.028

Woelbert, E., Kirtley, A., Balmer, N., Dix, S. (2019). How much is spent on mental health research: developing a system for categorising grant funding in the U.K. The Lancet Psychiatry, 6(5), 445-452. Doi: https://doi.org/10.1016/S2215-0366(19)30033-1

Yatham, L. N., Mackala, S., Basivireddy, J., Ahn, S., Walji, N., Hu, C., . . . Torres, I. J. (2017). Lurasidone versus treatment as usual for cognitive impairment in euthymic patients with bipolar I disorder: a randomised, open-label, pilot study. Lancet Psychiatry, 4(3), 208-217. doi: 10.1016/S2215-0366(17)30046-9

Yildiz-Yesiloglu, A., & Ankerst, D. P. (2006). Neurochemical alterations of the brain in bipolar disorder and their implications for pathophysiology: a systematic review of the in vivo proton magnetic resonance spectroscopy findings. Progress in Neuro-Psychopharmacology & Biological Psychiatry, 30(6), 969–995. http://doi.org/10.1016/j.pnpbp.2006.03.012

Yüksel, C., & Ongur, D. (2010). Magnetic resonance spectroscopy studies of glutamate-related abnormalities in mood disorders. Biological Psychiatry, 68(9), 785–794. http://doi.org/10.1016/j.biopsych.2010.06.016

Zalesky, A., Fornito, A., Cocchi, L., Gollo, L. L., van den Heuvel, M. P., & Breakspear, M. (2016). Connectome sensitivity or specificity: which is more important? NeuroImage, 142, 407–420. doi: https://doi.org/10.1016/j.neuroimage.2016.06.035

Zalesky, A., Fornito, A., Harding, I. H., Cocchi, L., Yücel, M., Pantelis, C., & Bullmore, E. T. (2010). Whole-brain anatomical networks: Does the choice of nodes matter? NeuroImage, 50(3), 970–983. doi: https://doi.org/10.1016/j.neuroimage.2009.12.027

Zhang, J., Wang, J., Wu, Q., Kuang, W., Huang, X., He, Y., & Gong, Q. (2011). Disrupted brain connectivity networks in drug-naive, first-episode major depressive disorder. Biol Psychiatry, 70(4), 334-342. doi: 10.1016/j.biopsych.2011.05.018
